# Drivers use active gaze to monitor waypoints during automated driving

**DOI:** 10.1038/s41598-020-80126-2

**Published:** 2021-01-08

**Authors:** Callum Mole, Jami Pekkanen, William E. A. Sheppard, Gustav Markkula, Richard M. Wilkie

**Affiliations:** 1 School of Psychology, University of Leeds, Leeds, UK; 2 Cognitive Science, University of Helsinki, Helsinki, Finland; 3 Institute for Transport Studies, University of Leeds, Leeds, UK

**Keywords:** Human behaviour, Navigation, Saccades

## Abstract

Automated vehicles (AVs) will change the role of the driver, from actively controlling the vehicle to primarily monitoring it. Removing the driver from the control loop could fundamentally change the way that drivers sample visual information from the scene, and in particular, alter the gaze patterns generated when under AV control. To better understand how automation affects gaze patterns this experiment used tightly controlled experimental conditions with a series of transitions from ‘Manual’ control to ‘Automated’ vehicle control. Automated trials were produced using either a ‘Replay’ of the driver’s own steering trajectories or standard ‘Stock’ trials that were identical for all participants. Gaze patterns produced during Manual and Automated conditions were recorded and compared. Overall the gaze patterns across conditions were very similar, but detailed analysis shows that drivers looked slightly further ahead (increased gaze time headway) during Automation with only small differences between Stock and Replay trials. A novel mixture modelling method decomposed gaze patterns into two distinct categories and revealed that the gaze time headway increased during Automation. Further analyses revealed that while there was a general shift to look further ahead (and fixate the bend entry earlier) when under automated vehicle control, similar waypoint-tracking gaze patterns were produced during Manual driving and Automation. The consistency of gaze patterns across driving modes suggests that active-gaze models (developed for manual driving) might be useful for monitoring driver engagement during Automated driving, with deviations in gaze behaviour from what would be expected during manual control potentially indicating that a driver is not closely monitoring the automated system.

## Introduction

Automated vehicles (AVs) are set to change the future of driving. Many AV systems will result in a change in the primary task when driving from manual steering control to vehicle monitoring. Whilst this change should eventually make driving safer^1^, there is currently a limited understanding of the impact of automation on human behaviour and capabilities when driving. An important aspect of human behaviour during driving, that may influence safety upon taking-over control, is where the driver is looking^2–4^. Driver gaze behaviour during automation appears to differ from manual driving (^5–7^, but see^8^ for a review), but the reason behind gaze changes remains unclear. To better understand the impact of automation on driver behaviour the present study examines gaze behaviour in detail, with a particular emphasis on how and why gaze changes between manual and automated steering control.

There is substantial evidence describing the active gaze behaviours produced during steering control: the majority of the time, when driving along curved roads, gaze is directed to a region ahead of the driver^9–12^. The time-window of this region is often stated to be 1-2 s^6,8,10,13–15^, based on empirical data from steering tight bends^10^ and slaloms^9^, as well as theoretical models^16,17^, but a distribution of 1-3 s accounts for a wider set of conditions including slower locomotor speeds (i.e. less demanding steering requirements,^11^) and individual variation^12^. The reason for gaze being directed to this time-window appears to be tightly bound to the current steering requirements. Changes in steering trajectory are accompanied by changes in gaze direction^18–21^ and changes in gaze direction usually result in changes to steering^22–25^.

**Figure 1:**
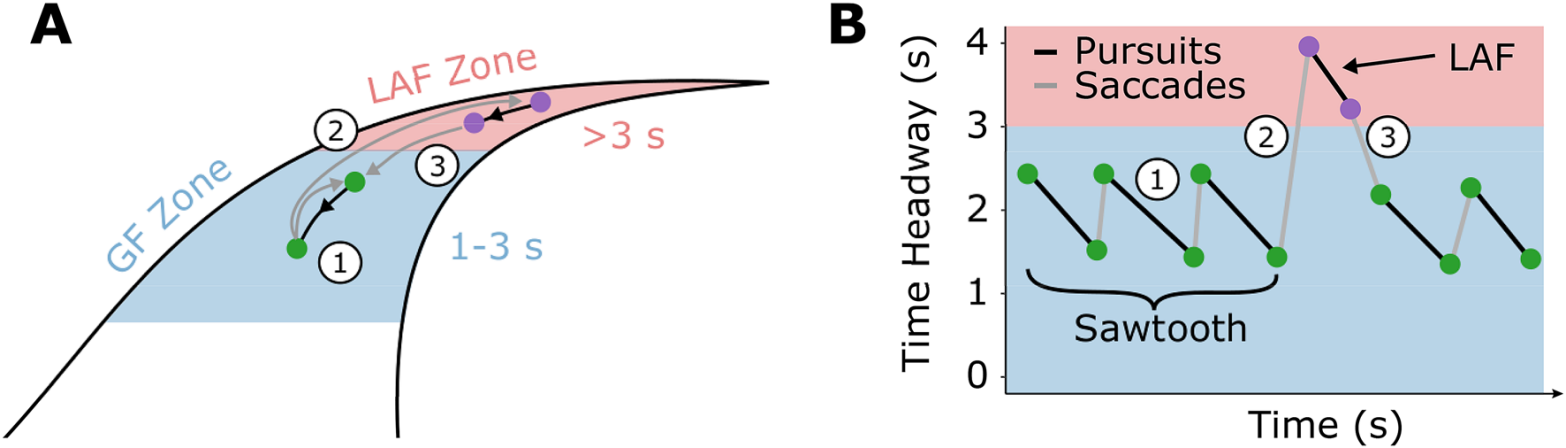
Gaze behaviour during curve driving. (**A**) When actively steering drivers spend most of the time looking at a ‘guiding fixation’ (GF) region 1-3 s ahead, with the occasional look-ahead fixation (LAF) further ahead. Within these zones, drivers pursue (track) waypoints using a move-dwell-move eye-movement pattern: drivers saccade to a waypoint, pursue (track) this waypoint, then saccade to a new waypoint. (**B**) The resulting gaze signal typically resembles an opto-kinetic nystagmus ‘sawtooth’ pattern, interspersed by infrequent lookahead fixations.

A functional role has been attributed to this observed relationship (for in depth reviews see^13,26^). In the literature examining the visual-motor control of steering, the functional role has been most commonly tied to the *time headway* of gaze: the time it would take to locomote to the point in the world where gaze falls^13^. Theories of the visual control of steering (derived from a broad literature base crossing vision science and engineering) suggest that points that are farther away in the visual scene will have different informational characteristics than closer points (e.g.^27^). According to the literature, gaze time headway should affect steering control such that gaze directed closer to the driver will lead to more precise steering (i.e. less positional error) but at the expense of steering smoothness^15,16,28^. The empirical data has been accompanied by models of steering control that use perceptual inputs that are sampled primarily from within the 1-3s time window^9,16,27,29–31^. The implication is that gaze fixations that land within the this time window serve an important role in obtaining the requisite information for controlling one’s trajectory, earning these fixations the label “Guiding Fixations” (GF; Fig. 1).

Whilst GFs appear to be crucial to the successful control of steering, drivers also exhibit gaze behaviours that fall beyond the time headway associated with GFs (i.e. >3 s). Gaze fixations to further points are often referred to as “Lookahead fixations” (LAFs), and have been associated with anticipatory decisions that affect the trajectory over longer timescales than the immediate control of steering actions (e.g. monitoring future decision points or obstacles;^14,32–34^). For a more complete consideration of the distinction between GFs and LAFs see (^13^, p. 989) and^6^.

It has been proposed that active steering through complex road geometries would typically be supported by drivers interleaving GFs (for supporting trajectory control) with LAFs (for retrieving anticipatory information; Fig. 1A). The question then arises: how are these patterns affected by vehicle automation when the human is no longer in manual control of the vehicle trajectory? During automation, some studies have reported a redistribution from GFs to LAFs^6,32,35^. Schnebelen et al.^6^ found that the proportion of gaze fixations directed beyond an empirically-identified LAF threshold increased during active steering, from 8.4% to 17.7% during the bend approach and from 4.5% to 32.3% during cornering. The functional distinction ascribed to GFs and LAFs during *manual* driving may help understand why the gaze behaviour appears to change during *automation*: perhaps drivers no longer need to prioritise perceptual inputs from the GF region for trajectory control, and so instead prioritise retrieval of anticipatory information from more distant regions^6,32,36^.

Whilst very few papers in the automation literature consider the GF/LAF distinction when examining gaze behaviour, many studies report gaze behaviour to be more variable or disordered during automation^5,7,36,37^ or less concentrated on the road ahead^2,38–41^. These findings describe very high-level changes to gaze and the measures potentially encompass a wide variety of eye-movement types that could be related to several task components, so it would be unwarranted to infer that more variability necessarily means more anticipatory (LAF) fixations. However, both increased gaze variability and reduced time looking at the road ahead can be contrasted with the tightly focused gaze patterns observed during high-speed manual driving (e.g.^11^), reinforcing the suggestion that GFs may be diminished during automation. Reduced numbers of GFs allow for an increase in the proportion of gaze patterns that serve other functions (e.g. anticipation^6^). However, it is important to note that even in these conditions GFs may still remain the predominant gaze behaviour—e.g. in Schnebelen et al.^6^ 70-80% of gaze data were classed as GFs.

To date there has not been any research examining changes to gaze patterns in the GF region during automation. The main reported metrics of gaze behaviour during automation have been based upon relatively coarse measures aggregated over time: e.g. variability^5^ or proportions of gaze allocated to different scene regions^38^. While these high-level descriptions can be useful, they stand in contrast with the fine-grained descriptions of the time-course of gaze patterns that are produced during active steering. A key characteristic of active steering gaze seems to be the ‘move-dwell-move’ sequence, consistent with an oculomotor pattern of tracking points that lie along the future path^9,11,42^. Recently, Tuhkanen et al.^11^ detailed the sequencing of gaze to a series of ‘waypoints’: drivers first generate a saccade to look at a point in the world 1-3 s in the future (speed dependent), then the eyes track this point for around 0.4 s, before the next saccade is generated to a new waypoint on the future path^11^. When these move-dwell-move patterns remain within the guiding fixation region for an extended period (i.e. there are few LAFs or other glances beyond the GF region) the vertical gaze angle trace resembles a ‘sawtooth’ pattern (also called opto-kinetic nystagmus^42–44^). Though the precise parameters for how far ahead the saccade lands and the duration of tracking may vary according to the task, the sawtooth pattern itself appears to be a common gaze behaviour produced during curve driving (^9,11,45,46^; Fig. 1B).

It has not been investigated whether the sawtooth gaze pattern observed during manual steering is present during automated steering, yet the possibility that this can be produced independently of manual steering control is of theoretical interest. During manual driving, there is evidence that the forward saccade (of the sawtooth pattern) is somewhat predictive and linked to the required steering^11^. In^11^, drivers looked at the location of a missing waypoint (embedded within a sequence of visible waypoints) in a manner that was coordinated with their steering actions. This empirical observation supports the theory that sequencing gaze onto waypoints will be linked to steering control^13^. Wider research into gaze allocation during visuomotor tasks also suggests that gaze is driven primarily by top-down mechanisms rather than the visual stimulus per se^47–53^. If sequencing gaze onto waypoints is predominantly related to the active control of steering, then this gaze pattern should be reduced/absent during automated vehicle control.

However, there is contrasting work suggesting that when observers view dynamic visual scenes without active control (i.e. videos, which are loosely analogous to our automated driving conditions) gaze behaviour is strongly determined by motion in the visual stimuli, rather than top-down factors such as task instructions or different mental models of a scene^54–58^. When driving a vehicle, eye-movement patterns during waypoint pursuit often appear to follow the motion of optic flow^42,46^. Given that at least some aspects of gaze behaviour seem to be produced as a result of the visual signals generated by self-motion through the world, there may be few differences between the gaze patterns produced when the visual stimuli are kept identical but drivers are simply no longer in active control of steering.

### Aims of the study

This manuscript aims to provide a detailed examination of how gaze patterns change between manual and automated vehicle control. There will be an emphasis on measuring gaze with respect to the GF regions that are associated with trajectory control during active steering. Whilst the relationship between active gaze and active steering is clear, there is little evidence to show that active steering is a prerequisite of producing those gaze patterns. During active steering, gaze could be driven both *predictively* from the required steering^11^ and *reflexively* from the incoming visual stimulus^54–58^. To the best of the authors’ knowledge, in all previous studies of gaze behaviour when driving automated vehicles, comparisons of manual and automated modes have altered *both* the need to actively steer (the *predictive* component) and the trajectories taken by the vehicle (which changes the *visual stimulus*), so changes to gaze patterns could be due to differences in action, perceptual signals, or both. The present study aims to decouple active control from perception to understand the influence of active control on gaze changes during automation. Highly controlled repeated conditions with matched visual conditions are used to isolate and identify the effect of removing the need to actively control steering on gaze behaviours.

To assess the extent to which active steering influences gaze behaviors, gaze patterns while driving around a virtual test track (“Manual” steering) can be compared with the patterns elicited by being driven by an automated vehicle (“Automated” steering). If Manual and Automated steering followed different trajectories, then this could also alter gaze patterns, so as to avoid this potential confound automated vehicle trajectories will be kept identical by replaying the participant’s own Manual trajectories (recorded earlier in the experiment; condition labelled “Auto-Replay”). Since the viewed trajectories in Manual and Auto-Replay will be generated by the same internal model (the participant’s control system) and will have identical visual stimuli, some similarity between gaze behaviours across these conditions may be expected. Therefore, Auto-Replay trials will be compared with pre-recorded “stock” trajectories (condition labelled “Auto-Stock”) to assess whether gaze similarities/differences (comparing Auto-Replay and Manual) are due to viewing identical trajectories produced by the drivers’ own internal model.

Given the importance of gaze time headway in modelling visual control of steering (e.g.^16^), time headway of gaze will be the key metric of interest. Though a number of papers have reported that drivers look further ahead when not in manual control of steering^6,32,59^, gaze direction has generally been reported in angular units (rather than scene-specific units such as time headway) or more coarse metrics (e.g. proportions of LAFs) that could overlook subtle changes in gaze patterns that nevertheless have implications for steering control after manual takeover. Gaze time headway distributions are characteristically right-tailed due to the occasional presence of LAFs to distant points^6^. Previous research has attempted to separate GFs and LAFs by defining a threshold beyond which a gaze fixation is categorised as a LAF^6,14,33^. However, a thresholding approach forces a binary classification onto gaze fixations as GFs or LAFs, as well as not accounting for road geometry and individual variation (see SI Appendix 1 for further discussion). In the current manuscript, gaze is decomposed using a mixture modelling approach that specifically ties gaze to road geometry, allowing for individual variation, and assigns probabilities rather than using discrete classifications. If successful this approach would seem to have the potential for scaling to other scenarios as outlined in the Discussion. As well as comparing gaze time headway measures, the presence of the sawtooth waypoint tracking gaze pattern will be examined across driving modes to determine whether these characteristic gaze behaviours remain intact.

This study will be the first to isolate the effect of removing steering control during automated driving upon gaze behaviours. The following detailed examination of gaze behaviour required the development of a novel method of analysing gaze patterns which used mixture models to separate GFs from potential LAFs.

## Results

### The experiment

**Figure 2:**
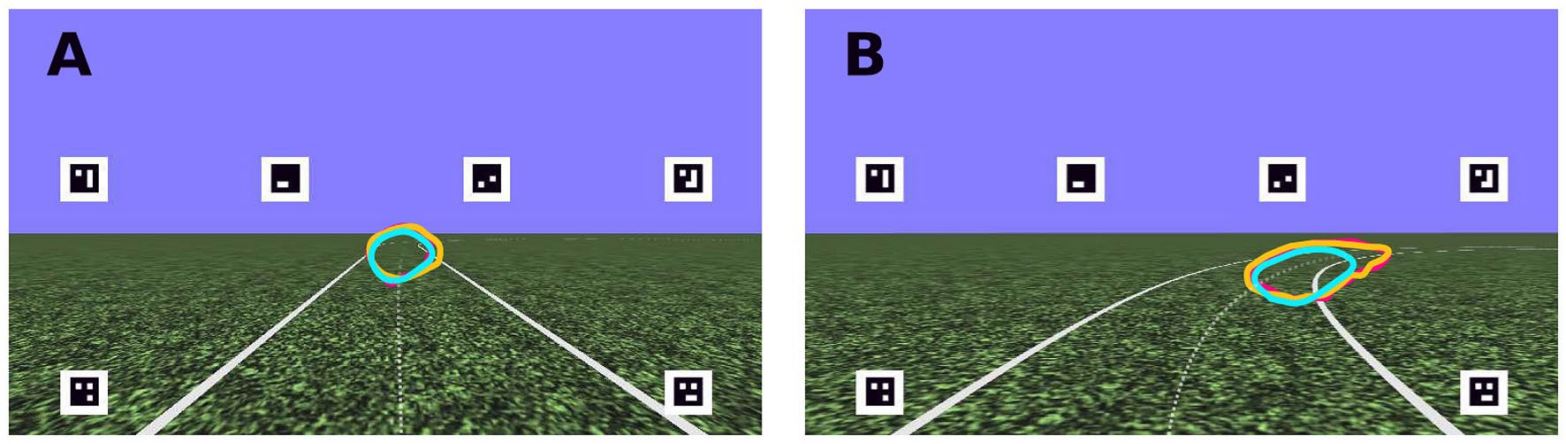
Screenshots. Screenshots of the straight (**A**) and bend (**B**) sections of the track, with gaze density rings superimposed to identify general regions of interest: the regions where 80% of gaze fell for Manual (cyan), Auto-Replay (yellow) and Auto-Stock (magenta) driving conditions. The track midline reference (dashed line) used for estimating gaze time headway (see Fig. 4) is the path equidistant to either road edge. The dashed midline was invisible during the experiment.

The experiment was conducted in a fixed-base driving simulator, with stimuli back-projected onto a large display (field of view 89$^{\circ }$ x 58$^{\circ }$), with black surroundings. The projected scene was a simple grass-textured ground-plane with super-imposed white lines demarcating the road^28^, excluding unnecessary scene elements that could potentially serve as irrelevant gaze fixation targets (Fig. 2). Five square optical markers were rendered on the display bordering the scene stimuli to support eye-tracking and localise the display surface for transforming gaze positions into screen coordinates (see “Methods” section). The road layout was an extended oval (Fig. 12), consisting of two 120 m straight sections followed by two bends of 25 m radius. The road width was 3 m and the speed of travel was constant at 8 m/s. Throughout the experiment, the driving mode alternated between periods of manual driving and automated driving, such that each full lap of the track was approximately half manual and half automation (see “Methods” section). There were three driving modes: Manual, where the participant had control of the steering wheel and the user inputs determined the direction of the vehicle; Auto-Replay, where the participant was driven around the track, and the trajectory was a replay of one of the participant’s own previous trials, with the visual signal and wheel motion fully automated; Auto-Stock, where the participant was driven around the track, and the trajectory was pre-recorded but not linked to the performance of the participant in the Manual condition. In the Auto-Replay and Auto-Stock conditions drivers were instructed to “keep their hands on the wheel as if steering, ready to take-over”. For further details see “Methods” section.

### Approach to statistical inference

Bayesian methods were adopted to estimate the extent and reliability of differences between conditions. There were no constraints on the gaze strategy participants could adopt, so trial-by-trial variability was expected (and observed). To avoid overfitting this trial-by-trial variability, inferences were made on aggregated measures from each participant’s pooled gaze data. Point estimates and 95% highest density intervals (HDI; the region that the model predicts with 95% probability the estimate will fall) are reported to help estimate the magnitude and reliability of differences respectively. Where it aids the interpretation of the results, the proportion of the probability mass that lies above zero will be reported (though there is no need to adopt dichotomous logic about the presence or absence of an effect). The full specification of each model, including the details of the model fitting process, is given in SI Appendix 2.

### Global time headway comparisons between driving modes

**Figure 3:**
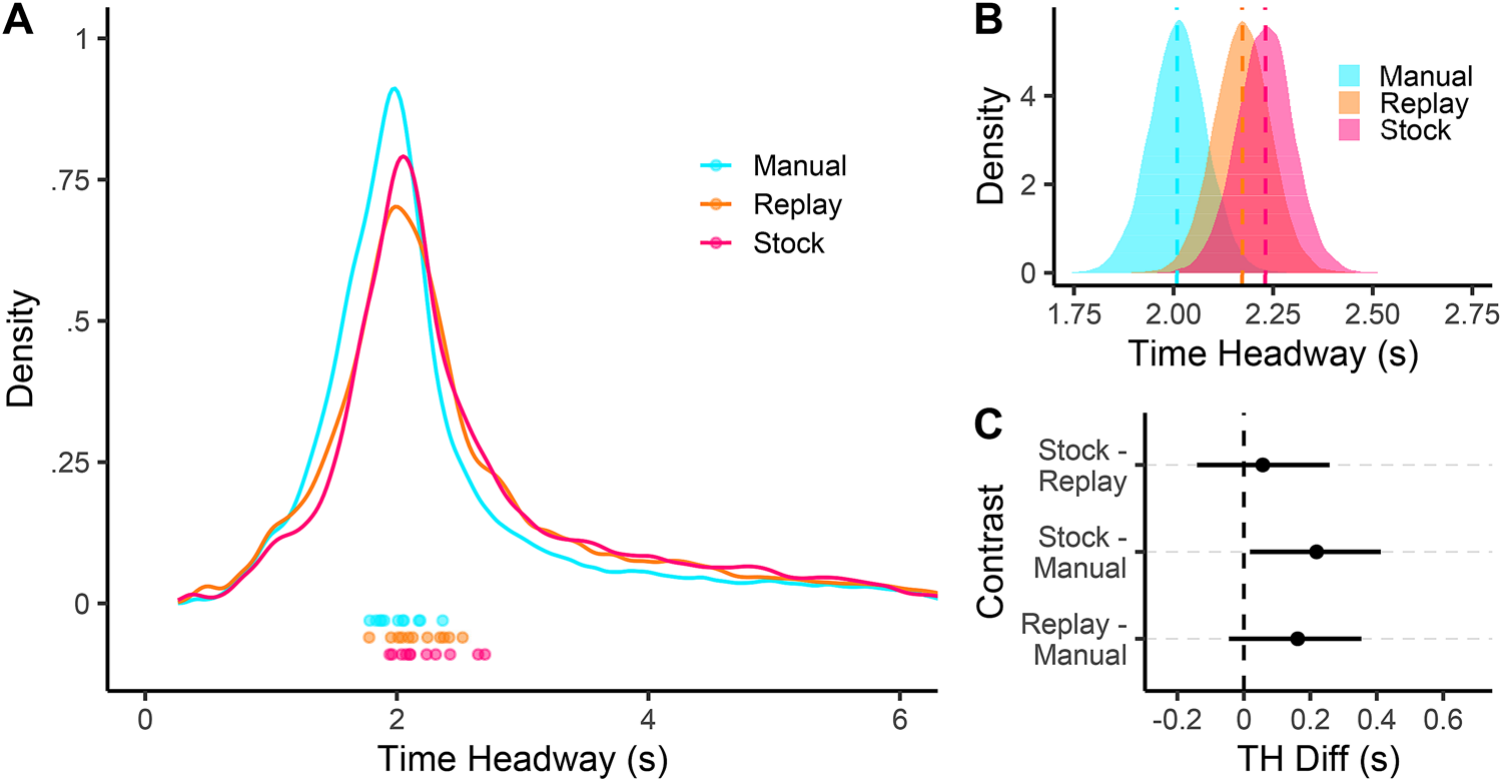
Time headway of gaze. (**A**) The distribution of pooled gaze Time Headway (TH) for each driving mode: Manual (cyan), Auto-Replay (orange) and Auto-Stock (magenta). At the bottom of the graph, the dots correspond to the individual participant’s median time headway for the three conditions. (**B**) The posterior distribution of the condition means (of participant medians) gaze time headway for each condition. Vertical dashed lines correspond to the empirical mean for each condition. (**C**) The posterior estimate of the differences between each condition. Means (dots) and 95% HDIs (bars) are shown.

A considerable analytic effort was taken to obtain a precise estimate of gaze time headway (see “Methods” section for further details): the time taken to move from the current vehicle position to the point in the world that gaze is directed toward, based on the current vehicle speed^13^. The first analysis examines high-level differences between the estimated gaze time headway signal (see *Estimating a Gaze Time Headway signal*) across the different driving modes. Figure 2 displays 80% gaze density regions, and shows that gaze sampling was broadly similar for Manual and Auto conditions. This finding is reinforced by the pooled gaze time headway distributions which were also similar across conditions (Fig. 3A). The Automated gaze time headway distribution appears to be shifted slightly later (rightwards) than the Manual conditions. The distributions are right-skewed, so participant medians were used when estimating condition time headway means across participants (Fig. 3B;^11^). The posterior estimates of the across-participant mean of medians for each condition are shown in Fig. 3B, overlaid with the empirical mean of medians (Manual mean = 2 s, SD = .18; Auto-Replay mean = 2.17 s, SD = .22; Auto-Stock mean = 2.22 s. SD = .26). Figure 3C shows the posterior distributions of the differences between driving modes. Both posterior contrasts for Auto-Replay (versus Manual) and Auto-Stock suggest that a non-zero difference is likely (for Auto-Replay 94.9% of the posterior mass is above zero; for Auto-Stock this value is 98.4%), but that any difference is likely to be small (Fig. 3C). Though the mean for Auto-Stock appears greater than Auto-Replay, the magnitude of the difference is estimated to be close to zero (72% of the posterior mass is above zero).

It seems, then, that drivers look slightly further ahead (by $\approx$  .2 s) during Automated driving compared to Manual driving. However, taking the central tendency of pooled time headway implies that all gaze patterns are functionally equivalent—even though recent research suggests distinct groupings of Guiding Fixations (GF) and Lookahead Fixations (LAFs; see Introduction for a full explanation of these classifications). Even though the uncluttered environment with simple, repeated road geometry could be classed as having little in the way of anticipatory information, gaze time headway distributions were consistently right-skewed (Fig. 3), which is characteristic of occasionally making LAFs^6^. With two functional groupings, there are three plausible and non-mutually exclusive reasons for the observed increase in time headway during automation: 1) time headway of GFs increase, 2) time headway of LAFs increase, or 3) fewer GF fixations are produced which leads to a greater proportion of LAFs. The next section examines whether the differences in Fig. 3 are caused by a change in GF behaviour or LAF behaviour (or both).

### Modelling gaze time headway as a mixture of separate components

This section provides a rationale for the analytic approach to separating GFs and LAFs. For technical specifications, see Supporting Information. Though the broad functional distinction between GFs and LAFs (GFs: immediate control; LAFs: anticipation) is theoretically useful, gaze function is difficult to ascribe on a fixation-level so care needs to be taken when separating different types of gaze. For example, in previous gaze decompositions^6,14,33^ ‘LAFs’ shared the commonality of *not being GFs*, but it is illogical that all gaze that is not GFs should share the same anticipatory functions sometimes ascribed to LAFs (indeed^6^ propose two functional sub-categories of mid-LAFs and far-LAFs). In the current experiment, we avoid potential misattribution of function by naming the non-GF gaze categorisation—which are *potential* LAFs but not *definite* LAFs in the anticipatory sense—after its corresponding road geometry (i.e. ‘Entry Fixations’, described later).

Previous attempts at separating gaze into components with different assumed functions (e.g. GFs and LAFs) have started with the assumption that the majority of gaze behaviours during curve driving consist of GFs^6,14,33^. On this basis, a measure of central tendency can act as a reference for GFs, and so gaze fixations that deviate from the central reference by some threshold value are categorised as LAFs^6,14,33^. The GF reference can either be determined *a priori*, using a point on the road with a time headway of 2 s^33^ or using an empirically driven value, such as the median eccentricity of the pooled gaze at each frame^6,14^. The LAF threshold value has previously been determined through researcher judgement^14^, or through fitting a mixture of Gaussian distributions to isolate a non-skewed central guiding fixation distribution, then taking the angular threshold at the tail of this distribution^6^.

A thresholding approach is useful for detecting gaze fixations that are clearly too far to be reasonably considered to be useful to direct steering control. There are, however, limitations with this method. A fixed threshold does not take into account variations in road geometry (which forces splitting data across some sections e.g.^6^) nor does it capture individual differences (c.f.^18^), and the choice of threshold value will bias resultant quantitative estimates of time headway. Furthermore, a fixed threshold forces a binary classification onto gaze patterns, which poorly reflects the functional significance of gaze fixations^60^. For example, a threshold with a generously high gaze time headway might mean that fixations exceeding the threshold are reliably classified as *not* GFs, yet the researcher is restricted to attributing equal functional significance to the remaining gaze data, which may not be wholly tied to trajectory guidance.

**Figure 4:**
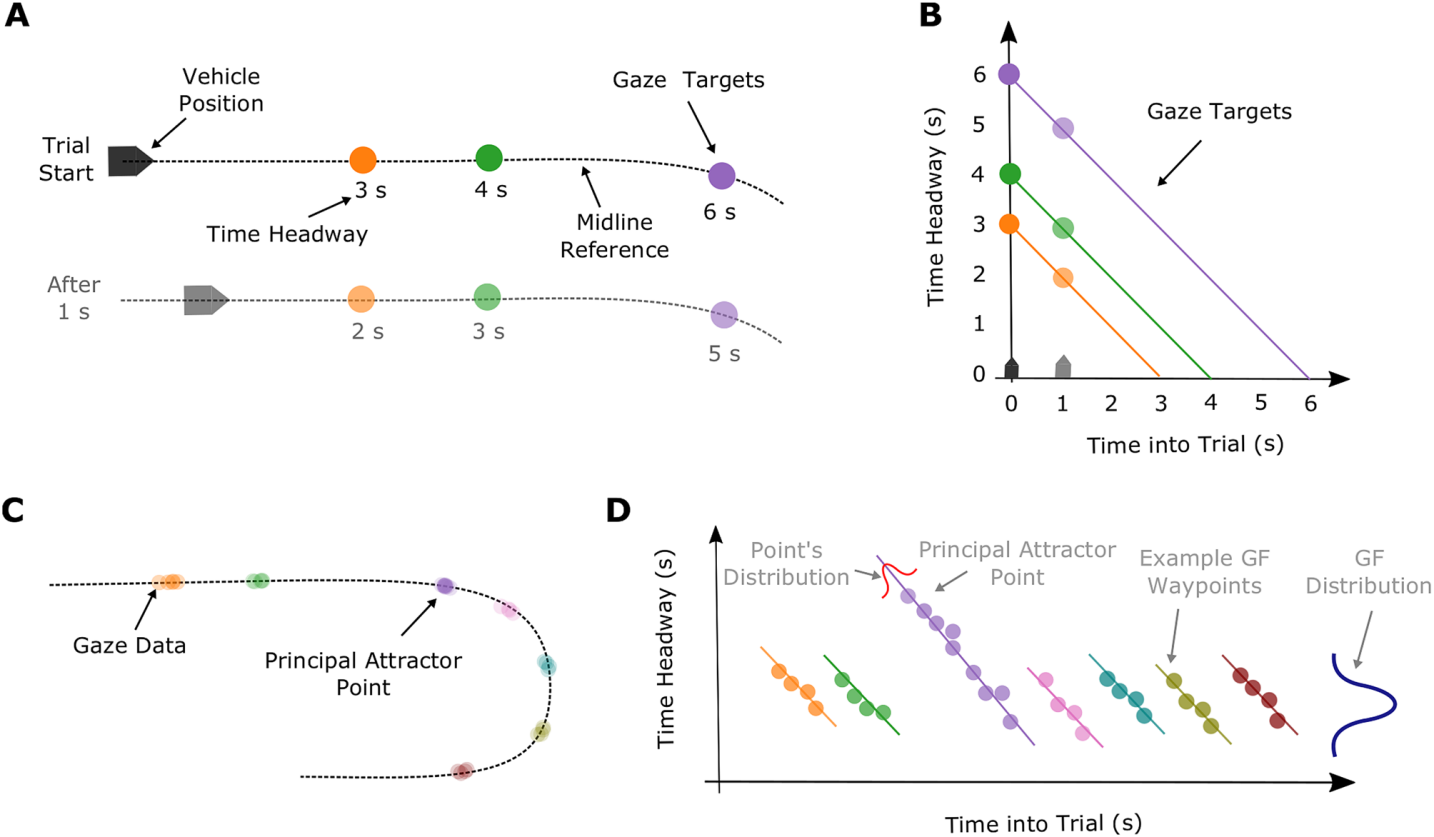
Gaze modelling. (**A**) Schematic top-down view of the waypoints along the midline reference (see Fig. 2) at two time points (0 s & 1 s into the trial). In (**B**) these two time points are plotted in the coordinate system of Time Headway of Gaze along the ordinate and Time into Trial along the abscissa. Each midline waypoint shown in A) represents a diagonal with a -1 slope in **B**). (**C**) Idealised gaze data for a trial shown in spatial (top-down) coordinates. The principal attractor point (purple) is fixated earlier and for longer than other, routine, waypoints. (**D**) Idealised gaze data from (**C**) shown in Time Headway x Time into Trial coordinates. The routine Guiding Fixation (GF) waypoints have a similar mean time headway and duration of fixation, so can be approximated by a normal distribution. On the other hand, the principal attractor point is not captured by the GF normal distribution so needs its own regression line.

Here we surmount the issues of using a fixed threshold value by using a novel mixture modelling method for decomposing gaze data. The modelling takes place in the coordinate system of *Gaze Time Headway* along the ordinate and the Drivers’ *Time into Trial* along the abscissa (Fig. 4B). Time into Trial refers to the distance, along the *midline reference* (Fig. 4A, see also Fig. 2), to the point on the midline reference that is closest to the driver’s current position in the world, converted into seconds. The measures of gaze time headway (see *Estimating a Gaze Time Headway Signal*) and time into Trial share a common reference of the midline (i.e. they are both in *time along midline* units). Therefore, if a driver fixates a point in the world and the vehicle continues to travel along the track (Fig. 4A), time headway of gaze will decrease at exactly the rate that Time into Trial will increase. In other words, gaze data associated with that fixation will have a slope of -1 (Fig. 4B). Each diagonal line, therefore, represents a point along the midline array that gaze could be mapped to (cf. *Estimating a Gaze Time Headway signal*). The y-axis intercept of each line is the total time along the midline (starting from zero at the beginning of the trial) of the gaze midline reference point (Fig. 4A&B).

It has been reported previously that drivers fixate successive waypoints *in the world* as they steer curved paths^11,42^. Example, idealised, gaze data (or more precisely the midline optical reference for each gaze landing point) for a trial is shown in Fig. 4C. Since each point in the world corresponds to a line with a slope of -1 in Fig. 4D, theoretically, every single fixation could be modelled by calculating an individual regression line for the corresponding midline array point. However, the goal of the current analysis was to decompose gaze into constituent parts, that is, to search for the clusters of gaze that share commonalities (and therefore presumably serve similar functions). We note that the bulk of gaze has previously been approximated by a normal distribution^6^. This approach appears valid since during curve driving the majority of gaze appears approximately normally distributed around a mean time headway^11^, and the *duration* for which drivers track each point is relatively constant^11^. Therefore, fixations that have a similar time headway (and tracking fixation duration) can be grouped under a normal distribution with a mean time headway and a standard deviation (Fig. 4D). In the coordinate system in Fig. 4D, a normal distribution on the marginal of time headway can be fitted by a regression line with a fixed slope of zero. Due to the independence from Time into Trial, these fixations can be thought of as falling within a region that moves with the observer.

Prolonged fixations to prominent allocentric points can be distinguished from possible GF candidates by having a gaze time headway that is larger or smaller than the primary bulk of fixations, also potentially with a tracking duration that is longer than is usual (Fig. 4C&D). These are objects or aspects of the road geometry that disproportionately attract gaze, called *principal attractor points*, which could potentially skew estimates of gaze time headway if unaccounted for.

**Figure 5:**
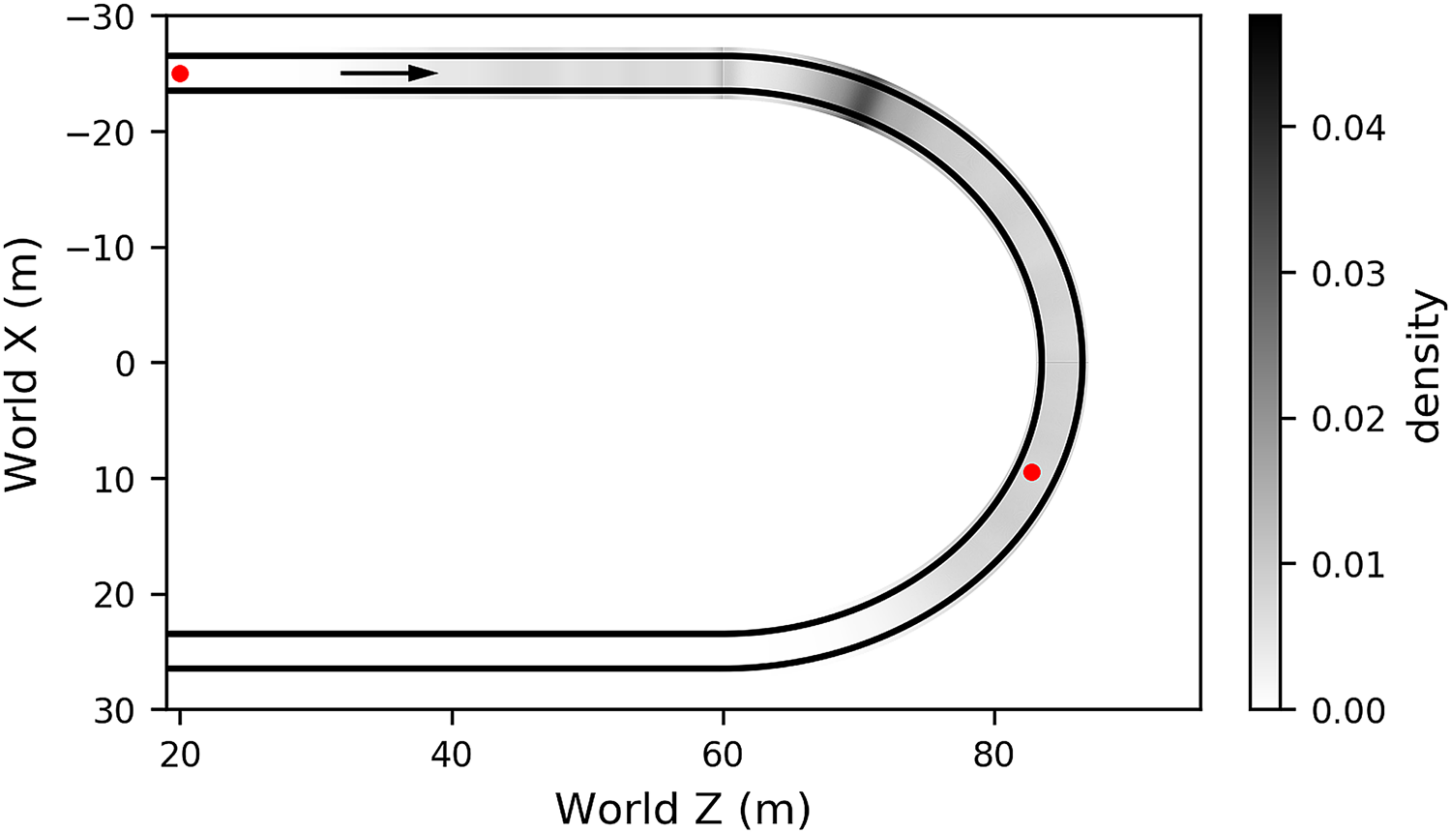
Gaze on track density. The density of the gaze midline reference (see *Estimating a Gaze Time Headway Signal*), pooled across participants. Note the spike in gaze density approximately 1 s after the bend entry. Red dots mark the vehicle position at the start and the end of the trial (i.e. the gaze data on the straight after the bend is excluded).

Given these principles, gaze time headway can be modelled as a combination of multiple regression models. A mixture modelling mathematical framework was used^61^, where gaze time headway is predicted from a weighted combination of different regression models. Each gaze observation is treated as having an associated probability vector denoting its correspondence to each regression model rather than a binary classification, bypassing the need for discrete threshold classifications. These methods captured the data well using only one principal attractor point: a section of the track just beyond the bend entry (Fig. 5), which meant that the mixture model only required two gaze clusters, ‘Guiding Fixations’ (GF) and ‘Entry Fixations’ (EF). Each cluster has a freely varying intercept and variance, so the model only has four free parameters. An additional noise cluster (with fixed parameters) was also included since the fitting process was susceptible to the inclusion of outliers that skewed these clusters (for further details see Supporting Information).

To allow for individual differences in GF and/or principal attractor point preference, the model is fitted separately to each participant’s pooled gaze data for each driving mode using the expectation-maximisation algorithm^62^. Since the final fit of the expectation-maximisation algorithm is sensitive to initial values we adopt a sparse grid of initial values and select the fit with the highest likelihood (for further details, see Supporting Information).

### Mixture modelling results

**Figure 6:**
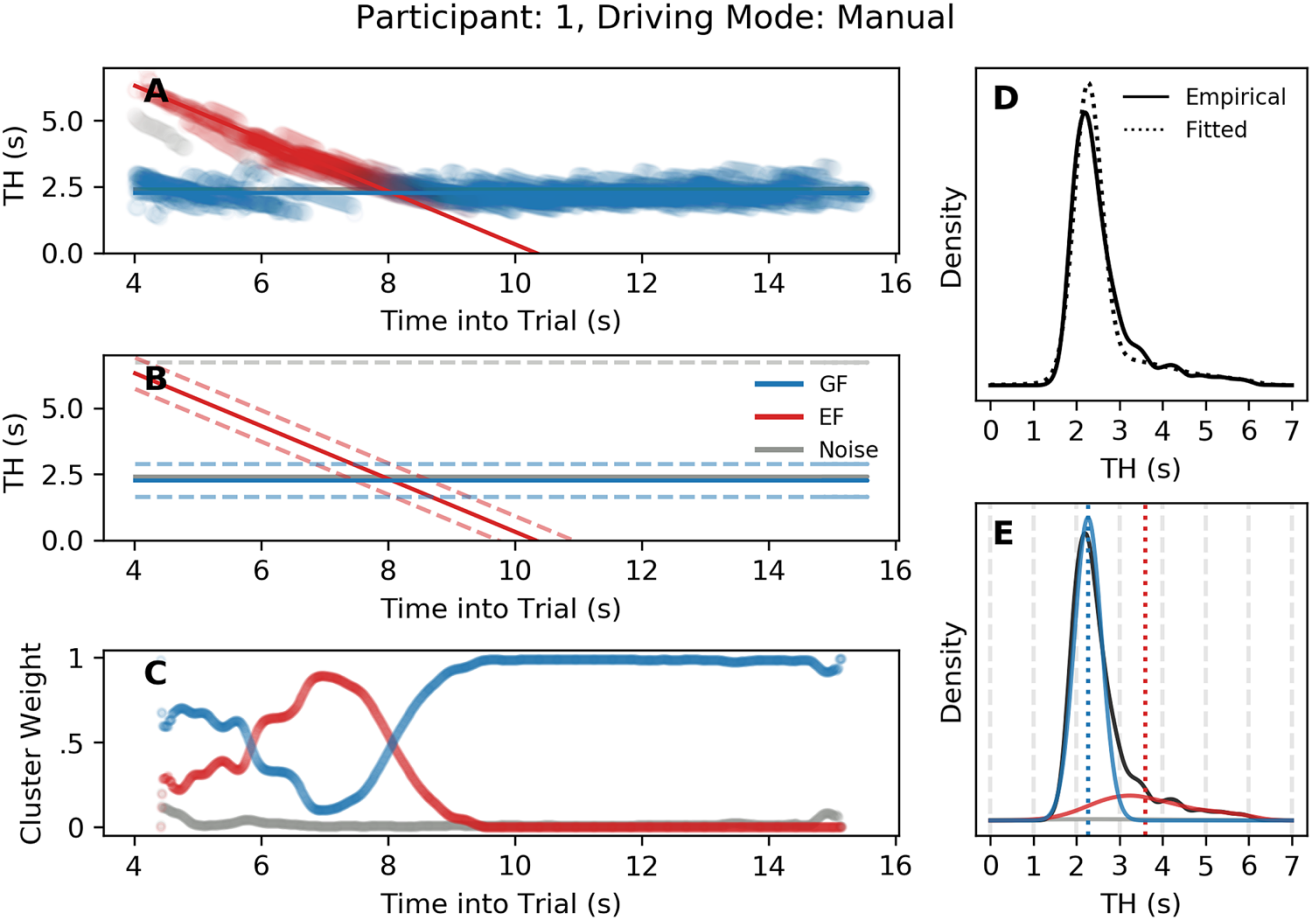
Model fit. A sample participant’s model fitting results. (**A**) Gaze data, with gaze Time Headway (TH) along the ordinate and Time into Trial along the abscissa. Gaze fixation data are shaded according to the probabilities of belonging to Guiding Fixations (GF; blue), Entry Fixations (EF; red) or Noise (Grey) clusters. (**B**) The regression lines and standard deviations of each model, from which a weighted sample is taken. (**C**) Smoothed cluster weights across the track. (**D**) Raw gaze TH density (solid black) overlaid with the fitted gaze TH density (dotted black). (**E**) The fitted gaze TH density decomposed into mixtures of GF (blue) and EF (red). Vertical dotted lines represent the estimated means.

A randomly selected individual’s fit is shown Fig. 6; all other individual fits can be found in Supporting Information. In some cases, noisy gaze data affects the fit, and it’s possible that some participants would benefit from including multiple additional principal attractor points. However, generally, the fitted density and empirical density match very well, and the restriction to one principal attractor point counters overfitting to noisy gaze data. The pattern observed in Fig. 6 is typical: the bend entry almost exclusively attracts gaze as one approaches it (see also the rise in weight attributed to the EF cluster in Fig. 6C), causing large Time Headways of 3–6 s, while throughout the bend gaze consistently falls within the 1-3 s region.

It is worth noting that it is tempting to associate the “Entry” cluster with lookahead fixations, since the bend entry has previously been reported as a key LAF target^14^. However, the cluster refers to a point in the world whereas LAFs are a broad spatiotemporal gaze category that could contain multiple points in the world. To emphasis this distinction the cluster is termed *Entry Fixation* (EF). Consider that a single prolonged entry fixation could start in the spatiotemporal zones ascribed to LAFs (Fig. 1); with high probability assigned to the EF cluster), continue through the GF region (with probability shared across GF and EF clusters), then reach closer time headways that would not fit the spatiotemporal descriptions of GFs or LAFs yet gaze probability would nevertheless be assigned to the EF cluster.

**Figure 7:**
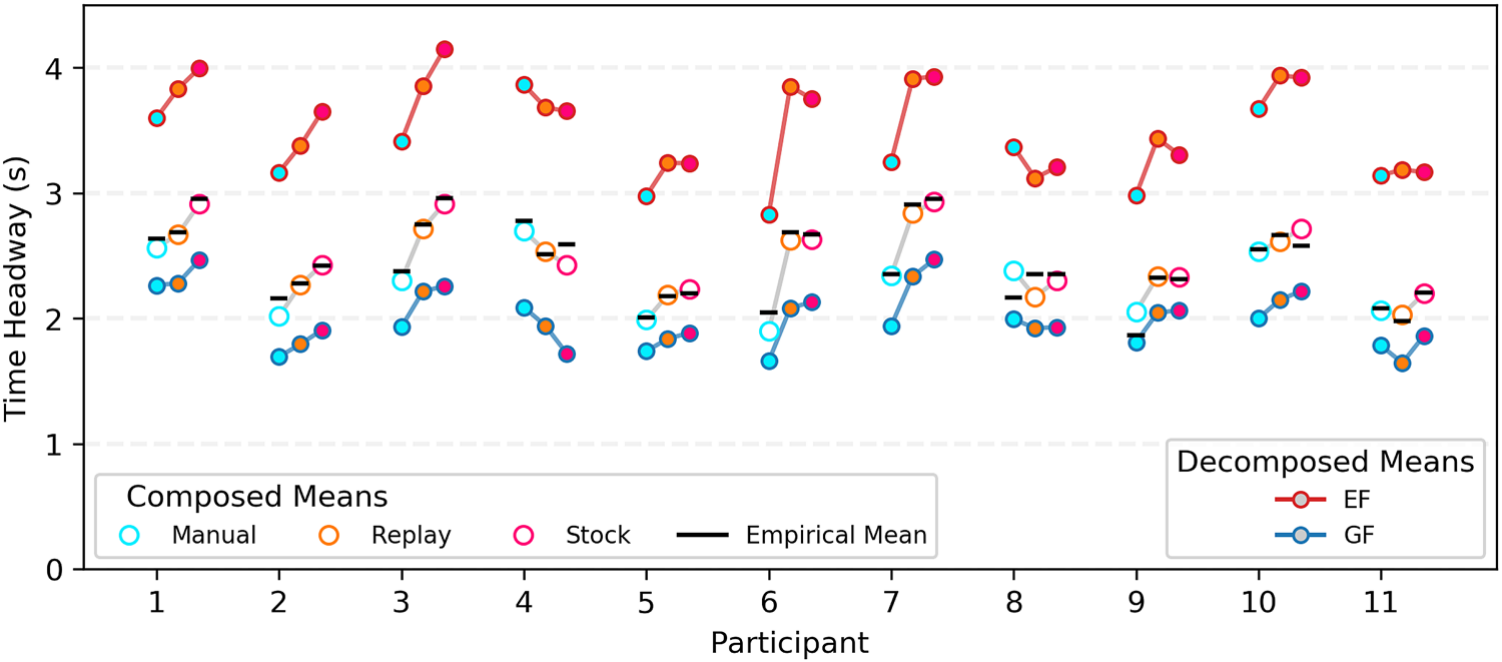
Individual means: Composed means, decomposed means, and empirical means for each participant. Larger circles are the mixture model composed means, coloured for each driving condition (Manual = cyan; Auto-Replay = yellow, Auto-Stock = magenta). Smaller circles and connected lines show the decomposed means for GF (blue) and EF (red). Black horizontal lines are the empirical means for that driving condition.

The fitted cluster means for all participants are shown in Fig. 7. The composed model mean closely matched the empirical mean (black) in most cases (mean absolute error = .06 s, SD = .06 s). For 8/11 participants there was an obvious positive slope across driving modes (Manual < Auto-Replay < Auto-Stock) for all estimated means (GF, EF, and Composed), showing that drivers consistently looked farther during Automated driving (for both EF and GFs). Two participants have clear negative slopes (4 & 8). Participant 8 was missing two blocks of data due to technical errors during data collection. Since the fitting process relies on regularities in the gaze data over a large number of samples, a smaller number of samples could bias the fit (see SI Fig. 11, SI Fig. 22, and SI Fig. 33). Participant 4 frequently glanced to near regions when not driving. In Auto-Replay (SI Fig. 18) these glances are distinctively directed to a small area (perhaps to the steering wheel; for other examples of this behaviour in different participants see SI Fig. 24, SI Fig. 34, and SI Fig. 35). In Auto-Stock these glances are less easily distinguished from the GF cluster (SI Fig. 29).

**Figure 8:**
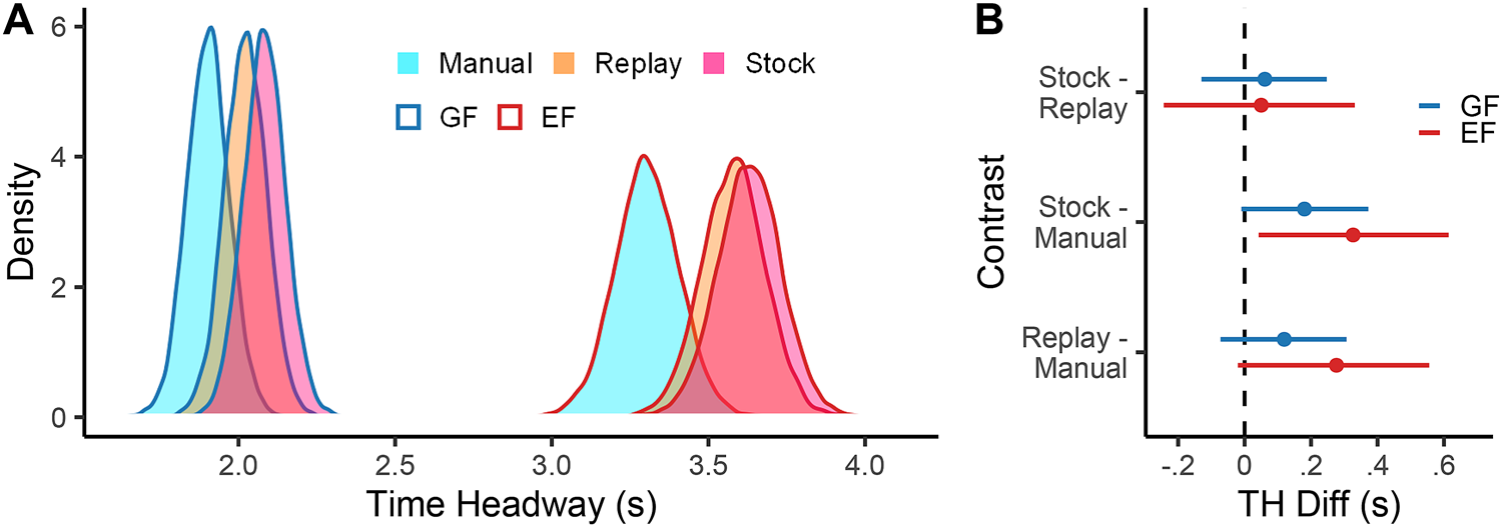
Posterior cluster means and contrasts. (**A**) Posterior distributions of condition means, for both GF and EF clusters. (**B**) Posterior estimates of the mean differences between each condition. Error bars are 95% highest density intervals.

A strong benefit of the mixture modelling approach adopted here is that clusters can be examined separately, rather than just studying the overall gaze distribution means. The posterior distributions for the cluster gaze time headway means are shown in Fig. 8A. The means for all driving modes are grouped closely around 2 s for GFs and 3.5 s for EFs, though for both clusters the means are smallest for Manual and largest for Auto-Stock. The contrasts shown in Fig. 8B indicate that for both GFs and EFs there is a high likelihood that the difference in gaze time headway between Manual and Auto conditions (the bottom two contrasts in Fig. 8B) is small and positive (i.e. During automation gaze falls slightly further ahead than Manual drivers). While the magnitude of the difference is estimated to be slightly higher for EFs (Auto-Replay - Manual = .25 s, [− .01, .51], 97%>0; Auto-Stock - Manual = .31 s, [.04, .57], 99% > 0), there is also more uncertainty around this estimate (GF contrasts are: Auto-Replay - Manual = .12 s, [− .08, .32], 89.5% > 0; Auto-Stock - Manual = .18 s, [− .02, .39], 96% > 0). Conversely, both cluster contrasts for Auto-Stock and Auto-Replay predict a difference of near zero. In other words, we can be confident that any effect between Auto-Stock and Auto-Replay conditions is only slight.

The increase in EF cluster gaze time headway for automation could be due fixating a farther away EF point for the same amount of time, fixating the same point for a longer time, or fixating the same point earlier, when the driver is farther away (or a combination of all factors). Supplementary analyses show that drivers fixated a similar EF point across conditions (see SI Fig. 2). The majority of gaze was assigned to GF cluster across all Manual (Posterior mean = .75 [.72, .77]), Auto-Replay (Posterior mean = .72 [.7, .75]), and Auto-Stock (Posterior mean = .7 [.68, .73]) conditions (see SI Fig. 3). The gaze probability assigned to GFs slightly reduces (and we observe a reciprocal increase in EF fixations) during automation (Auto-Replay - Manual contrast mean = − .02, [− .06, .02], 87.6% of the posterior mass < 0), and reduces slightly further when the trajectory is unfamiliar (Auto-Stock - Auto-Replay contrast = − .02, [− .06, .02], 86% of the posterior mass < 0). These analyses show that the increase in the EF cluster gaze time headway was partly due to fixating the bend entry for (marginally) longer, and partly due to fixating the bend entry earlier.

In summary, the analyses in this section replicate existing findings of increased overall lookahead during automated driving but provide a more detailed insight into how this overall increase occurs: a minor redistribution of gaze from GFs to EFs, combined with an increase of time headway for GFs and EFs, with an indication of the increase being larger for EFs than for GFs.

### Examining saccadic behaviour associated with waypoint sequencing

The previous analysis section showed that gaze is shifted only $\approx$.2 s ahead when active steering control is removed and the visual information is kept constant. This shift is apparent for both the bulk of GFs and also fixations at a longer time headway to the bend entry. In the previous section gaze is called ‘fixations’ to loosely refer to gaze that is directed to particular places. However, the analysis is conducted on the pooled time headway signal rather than isolating specific fixation events, where observers pursue a point in the world (i.e. a fixation). To determine where the shift in time headway occurs this next section examines the gaze signal in more detail.

**Figure 9:**
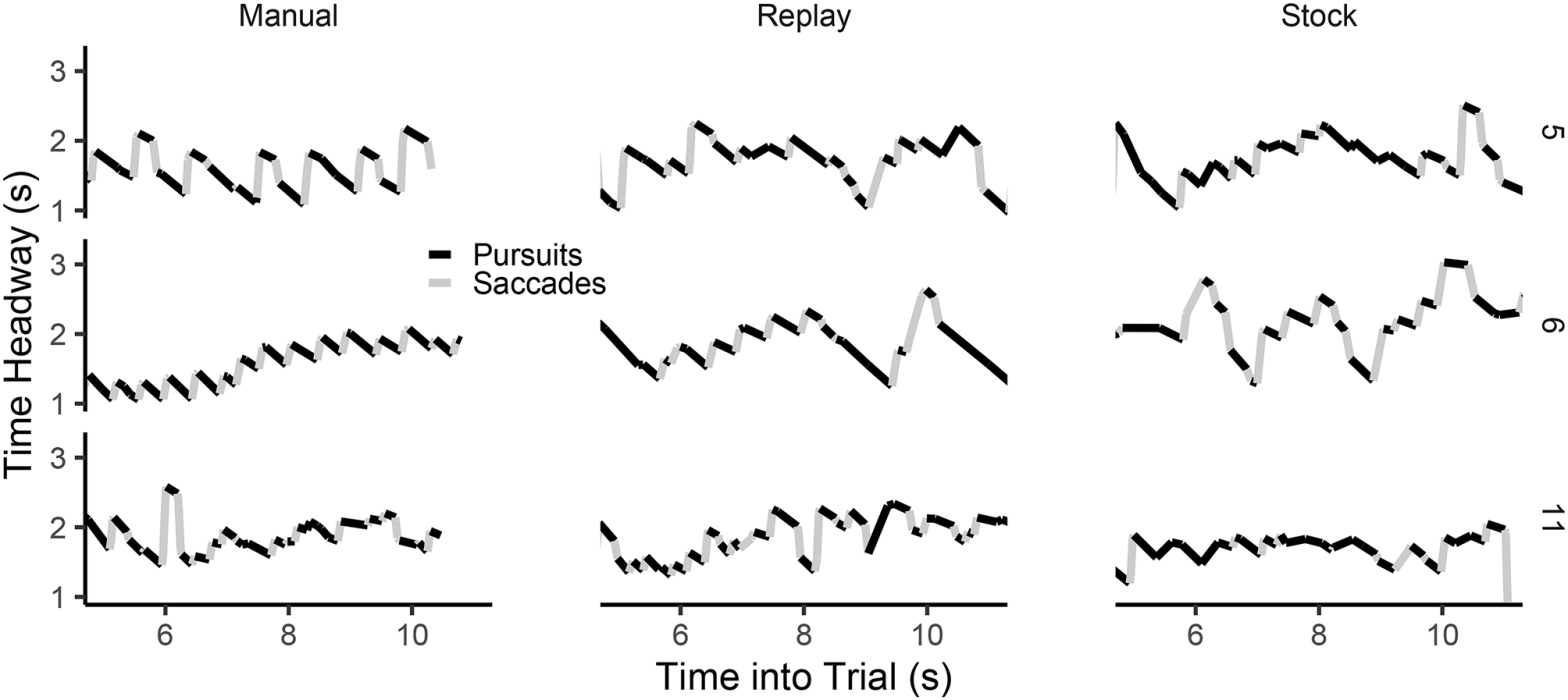
Example gaze traces. Three randomly selected participants (rows) and trials (panels) for Manual (left), Auto-Replay (the same trial replayed; centre), and a Auto-Stock trial (right).

During active steering, drivers tend to sequence gaze for fixating and tracking a series of successive waypoints (the sawtooth gaze pattern). Figure 9 shows example vertical gaze angle traces for three randomly selected participants during a Manual trial, the corresponding matched Auto-Replay trial, and an Auto-Stock trial. All examples in Fig. 9 contain repetitions of a sequence where the participant tracks a point, followed by a forward saccade, and then tracking a new point. There are individual differences in the consistency of this pattern, reflected in the duration of the pursuit, the forward-saccade amplitude, and the degree to which gaze ‘jumps’ from far regions (longer gaze time headway; e.g. an EF) to near regions (shorter gaze time headway; e.g. a GF), a phenomenon termed ‘gaze polling’ (cf.^18^). These examples illustrate well the heterogeneity in gaze patterns across different participants when examined at an individual level, despite similarities in other gaze measures. Waypoint tracking behaviour can be examined by assessing the time headway of where the forward saccade is launched from and where it lands, and also the duration over which each point is tracked^11^. The shift in time headway (described in the previous sections) could be caused either by drivers producing larger saccades that land farther ahead, or by not tracking points for as long.

To sequence gaze, we parsed the gaze signal into tracking eye-movements (pursuits and fixations) and saccades using naive segmented linear regression^63^. The original groups of pursuits and fixations were collapsed into the same class (for the present steering task both are considered tracking fixations), yielding classifications as illustrated in Fig. 9. This method allows us to report the gaze time headway of saccade launch-points and land-points, and inter-saccadic tracking duration^11^. For this analyses we restrict gaze data to when the driver position was on the bending portion of the track. By removing the beginning straight we avoid prolonged fixations to the bend entry confounding the waypoint tracking analysis, and instead focus on the GF region where active-gaze patterns have been rigorously described^9,11,42^.

**Figure 10:**
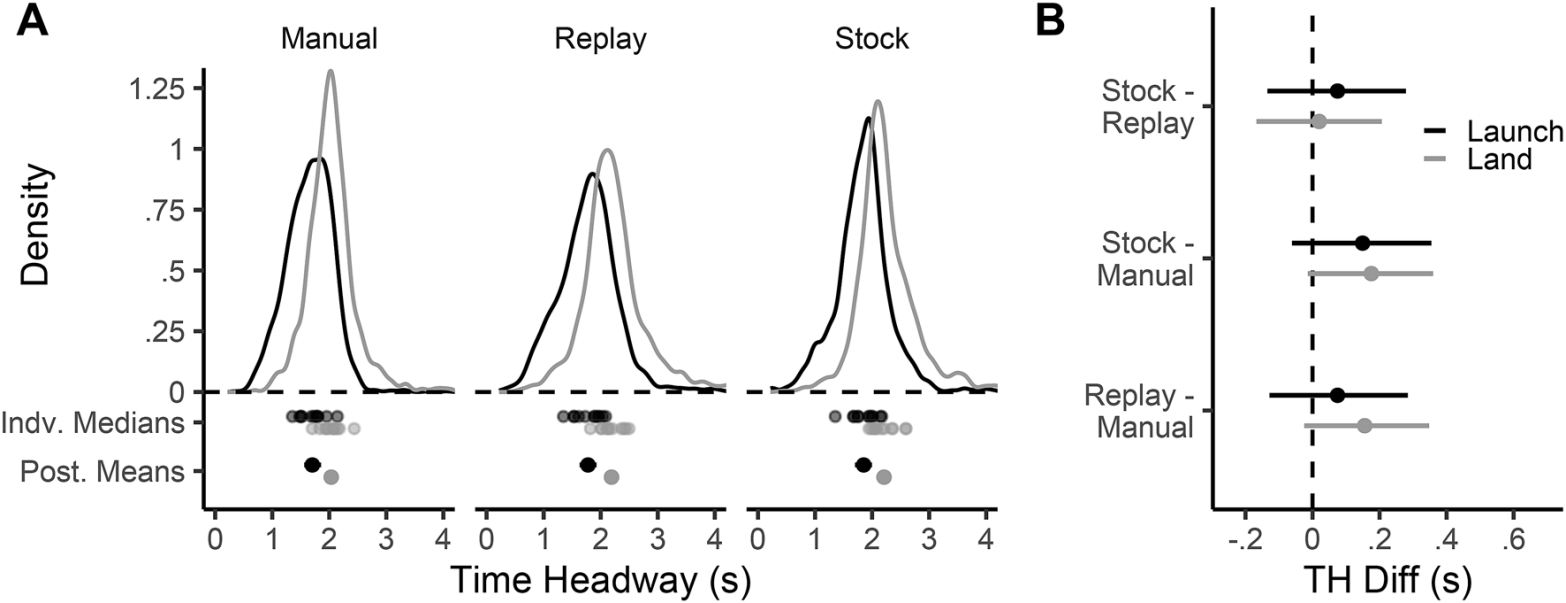
Saccade launch and land time headways. (**A**) The pooled empirical distribution of gaze Launch (black) and Land (grey) Time Headways (TH) for each driving mode. Also shown are the participant medians, and the posterior estimates of the mean of medians with 95% HDIs. (**B**) The Posterior estimate of the mean TH difference between conditions, with 95% HDIs shown, for saccade launch-points (black) and saccade land-points (grey).

The saccade launch and land time headways for forward saccades are broadly similar across driving modes (Fig. 10A with the participant launch points falling in the range 1.35–2.16 s, and participant land points falling in the range 1.7–2.6 s. The estimated condition contrasts for both launch and land time headways are shown in Fig. 10B. Both launch and land time headway increase slightly from Manual to Auto-Replay (and increase further in Auto-Stock), however, the estimated mean difference between Manual driving and the Auto conditions is more pronounced for saccade land-points (Auto-Stock - Manual = .18 [.0, .36], 97% > 0; Auto-Replay - Manual = .16 [− .02, .35], 95.3% > 0) than for saccade launch-points (Auto-Stock - Manual = .15 [− .05, .35], 92.4% > 0; Auto-Replay - Manual = .08 [− .14, .28], 76.6% > 0). The difference between Auto-Stock and Auto-Replay is estimated to be close to zero.

**Figure 11:**
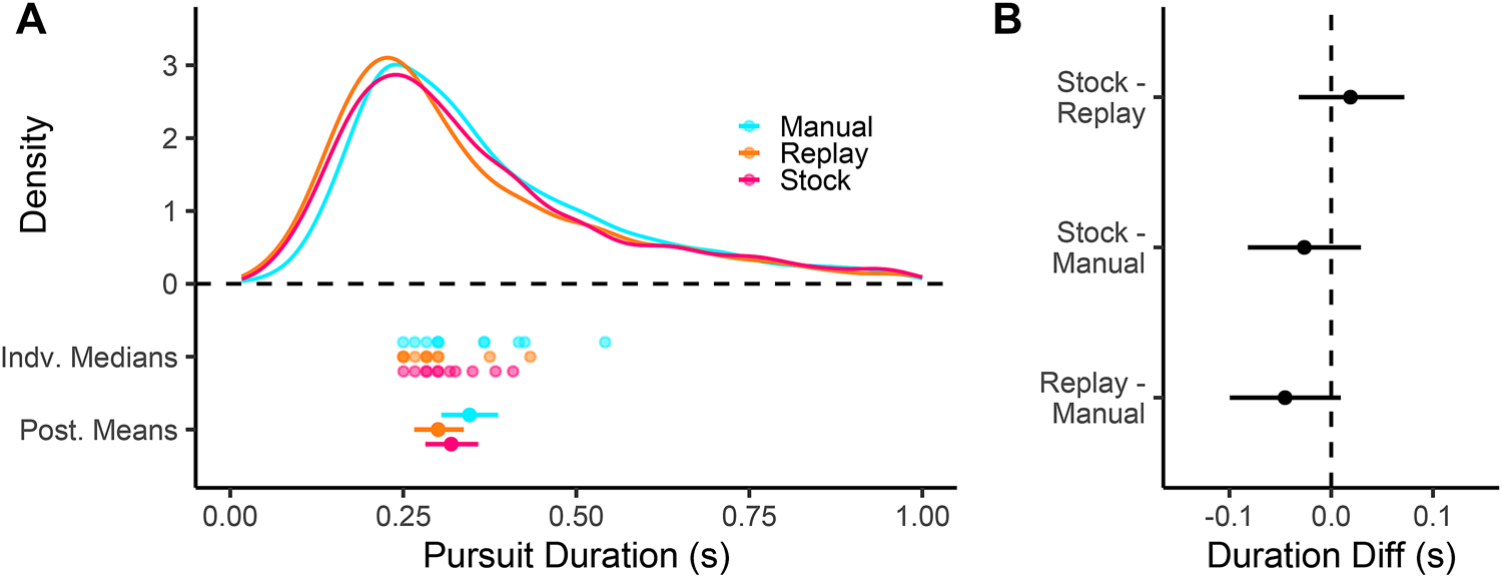
Gaze tracking duration. (**A**) The distribution of pooled gaze tracking duration, of Manual (cyan), Auto-Replay (orange) and Auto-Stock (magenta) conditions. Shown below are the participant medians, and the posterior estimates of the mean of medians. (**B**) The posterior estimates of the mean difference between conditions, with 95% HDIs shown.

The tracking duration is shown in Fig. 11A, alongside the individual medians and posterior estimates of condition means (of medians). The Manual pooled distribution is shifted rightward, indicating longer tracking durations, a pattern that is born out with a higher posterior mean (Manual posterior mean = .35 s [.31, .39]). The posterior estimate of the difference between means is shown in Fig. 11B. For both Auto-Replay and Auto-Stock the mean difference is predicted to be negative, meaning that Manual drivers tracked for longer. The magnitude of the difference, and the certainty that the effect is in the predicted direction, varies from Auto-Replay (Auto-Replay - Manual = − .05 [− .10, 0], 95.2% < 0) to Auto-Stock (Auto-Stock - Manual = − .03 [− .08, .03], 83.3% < 0). Again, the difference between Auto-Stock and Auto-Replay is estimated to be close to zero.

The gaze sequencing analysis shows that active-gaze patterns were present in all driving modes. During automation there was a small overall shift further ahead, and drivers tracked points for a shorter time, when compared to manual driving.

## Discussion

This experiment was designed to examine gaze behaviours during manual and automated driving under highly controlled conditions where the visual stimuli could be matched across driving modes, to isolate the effect of active steering control upon gaze behaviours. Drivers looked slightly further ahead during automation, yet monitored the scene with waypoint-tracking gaze patterns that were similar to those observed during active steering.

Using a novel mixture modelling method to decompose gaze, it was found that drivers were drawn to look toward the bend entry during all driving conditions and that these entry fixations could be separated from the more routine guiding fixations. For manual driving, the mean guiding fixation time headway was 1.91 s, which sits within the range of estimates previously observed during manual driving^10–12,15^. The entry fixation time headway during manual driving was 3.32 s, which would be classified as LAFs according to previously used thresholds, either methodologically (e.g.^33^) or anecdotally (e.g.^13^) (see *Introduction*). A small increase in gaze time headway during automation was observed for *both* guiding fixations and entry fixations shifting farther ahead (GFs by $\approx$.15 s, EFs by $\approx$.28 s). Since entry fixations were directed to a point in the world, the mean gaze time headway of this cluster is a product of where this point is located, and how early before reaching it the driver begins polling it. The average entry fixation placement was very similar across driving modes (see SI Fig. 2) so the increase in gaze time headway was primarily due to fixating the bend earlier. During automation drivers also fixated the bend entry for a *longer total time*, as shown by the slight decrease in guiding fixations and a concomitant increase in entry fixations. In general, then, automation caused the drivers to look farther down the road than during manual control of driving.

The sequencing of gaze was assessed to determine whether the shift in gaze time headway meant that the waypoint-style gaze tracking observed during manual steering control changed during automation. For manual driving, the mean estimated tracking duration (.35 s) is similar to the $\approx$.38 s reported in^11^ using a different course. The overall pattern of waypoint-tracking appeared to be preserved across driving modes, suggesting that drivers employ similar gaze strategies when monitoring locomotion as when actively steering. However, the entire gaze pattern was shifted farther ahead during automation (i.e. larger gaze time headway observed for both forward saccade launch and land points), and drivers tracked waypoints for shorter periods compared to during manual control.

Though there were some important differences between gaze behaviour during Manual and Automation conditions (discussed later), the most compelling aspect of these results is that that the gaze behaviours were qualitatively and quantitatively similar across conditions. Both Manual and Automated conditions led to similar waypoint tracking style gaze patterns, and the quantitative differences in gaze time headway are estimated to be small (in the region of .1–.3 s). The similarities suggest that the fundamental spatiotemporal characteristics of gaze—how far ahead one looks and how long a point is tracked for—are not substantially altered even when active control of steering is removed.

In some respects these similarities are surprising, since the amplitude and location of the forward saccades have previously been shown to correlate strongly with the required upcoming steering action^11^, and there is the theoretical rationale to suggest that the predictive information available from generating steering commands aids saccade planning (reviewed in^8,13^). The consistency of gaze patterns across Manual and Automation modes could indicate that gaze patterns are driven primarily by the apparent motion of the visual scene, so when the stimulus is identical (Auto-Replay) or even similar (Auto-Stock) to manual control conditions, then similar gaze patterns will be produced. Indeed, some aspects of gaze behaviour when viewing static scenes have been effectively captured using models relying on bottom-up processing^47,64,65^, and there is considerable evidence that gaze behaviour during video viewing is informed primarily by stimulus motion^54–58^. Therefore, one hypothesis consistent with the data is that the similarities between Auto-Replay and Auto-Stock are determined in a bottom-up manner from the stimulus motion, with the additional task of controlling one’s trajectory in the Manual condition causing gaze to move closer due to tighter gaze-steering coordination^11,13,18^. However, a purely stimulus-driven account of the observed similarities does not take into account that purely bottom-up models have fared poorly trying to capture gaze responses when interacting with dynamic scenes^47,53,65–68^. Instead, the primary driver of gaze allocation may be the task the observer was performing^47–50,53,66^. If the current task is the primary driver of gaze behaviour^51,52^, then in the present experiment, given the similarities between Manual and Automation conditions, it is worth examining what functions may be shared between these driving modes. In order to be useful for controlling a driver’s trajectory, guiding fixations need to support reliable estimates of self-motion^17,69^. Additionally, estimating the motion of the vehicle with respect to the environment is a core function of the monitoring task during automation, since executing the appropriate steering commands upon manual takeover (and detecting any automation errors) requires an accurate estimate of the current vehicle motion^8,70,71^. Estimating the vehicle’s trajectory, therefore, could be the task that is producing similar gaze behaviours across both driving modes.

If gaze similarities are due to the monitoring of self-motion, the question remains how can drivers produce such similar gaze patterns across Manual and Automation conditions, even though the predictive information given by active steering control is removed during Automation? To answer this question we propose two further hypotheses that are consistent with the current data. Firstly, it could be that a monitoring driver maintains an updated internal model of the automated vehicle’s driving behaviour^72,73^, which is then used to program the initial launch saccade, and then each new saccade is executed when the model’s state needs updating. A similar model-predictive account for saccadic behaviour during manual steering has been proposed^11,13^. Secondly, it could be that the approximate gaze time headway and waypoint tracking duration is extracted during periods of manual control, which then act as a template pattern for the monitoring performed during automation trials. In the second instance, the learned saccadic behaviour could supply *inverse models*^74,75^ that determine approximate saccade amplitude and location given the goal of monitoring the vehicle state. These hypotheses are not mutually exclusive, and it is possible that both mechanisms may play a role in the exhibited behaviours.

Despite the similar gaze patterns across conditions, there were also important changes during Automation: drivers shifted gaze slightly farther ahead and tracked points for shorter periods. The shift in gaze time headway to look further ahead during automation supports previous research in this domain^6,32,59^. However, previous studies have focused on an increase in the proportion of Lookahead Fixations (LAFs) to regions far ahead of the driver. While the present findings show that automation slightly increased fixations of the bend entry (which in the current study is the primary LAF candidate), this increase was small ($\approx 2\%$). Instead, the overall increase in gaze time headway seems to be primarily driven by a forward gaze shift of around 0.2 s, for both GFs and EFs. The functional basis of this shift would appear to be quite different from a simple increase in the proportion of LAFs. During manual driving, LAFs are assumed to support route choices that alter the course of the trajectory at a macro level (e.g. whether one needs to skirt an upcoming obstacle). In both Navarro et al.^32^ and Schnebelen et al.^6^ the driving tasks created a need to anticipate, either the location of obstacles^32^ or a sequence of unfamiliar bends (of varying radii and direction), in order to supervise an automated vehicle^32^. Increased LAFs during these driving tasks were presumably linked to the anticipatory function of these fixations. Conversely, in the current experiment, there was *less* need to anticipate: the road geometry was designed to be deliberately predictable (i.e. a simple and repeated track situated within a sparse environment) to reduce the need for anticipatory gaze behaviour, and the takeover point was consistent throughout the experiment. Though the increase in EF/LAF probability during automation *is* consistent with a shift towards anticipatory monitoring, the repeated road geometry and low risk may explain why this increase was only small.

The predictable driving task may also mean that the shifts in gaze time headway further ahead during automation cannot simply be attributed to anticipation, especially since shifts in gaze time headway occurred *within* the guiding fixation region itself (a region usually sampled to support trajectory control^6,9–13,16^). Rather than enabling anticipation per se, it could be hypothesised that the shifts in gaze time headway support the monitoring of the vehicle’s motion under increased uncertainty. Automation effectively removes the predictive signal provided during active steering which increases uncertainty when estimating the vehicle’s current trajectory. Additional preview might be useful to compensate for this increased uncertainty, resulting in an overall shift in time headway farther ahead when not in manual control.

The proposal that gaze behaviour changes are caused by increased uncertainty about the vehicle’s motion might also help explain two other intriguing aspects of the data. Firstly, drivers track waypoints for a shorter period during automation. It has been suggested that drivers sample objects more frequently if uncertainty about task-relevant visual information is introduced^48,49,76^, so if drivers are less able to predict the vehicle’s trajectory (i.e. there is more uncertainty) they might need to sample new waypoints more frequently. Secondly, on all measures, the difference between Auto-Stock and Auto-Replay was estimated to be close to zero but always *in the same direction* as the difference between Manual and Auto-Replay (meaning that the difference between Auto-Stock and Manual was usually the largest magnitude contrast). If the shift in gaze time headway from Manual to Auto-Replay was due to reduced ability to predict the vehicle’s trajectory (due to not actively steering), it follows that viewing a trajectory that is not generated by your own internal model (as is the case in Auto-Stock) should further reduce the ability to predict a vehicle’s trajectory. The notion of an additive effect, with *removing active steering* and an *unfamiliar trajectory* resulting in *cumulative* effects on gaze, is consistent with both the data and the increased uncertainty hypothesis outlined above, but needs to be fully tested in future research.

Irrespective of the reason underpinning the differences between gaze during manual and automated driving, there remains sufficient similarities in the basic patterns that active-gaze models developed for manual driving should have utility for monitoring and interpreting gaze behaviour during automated driving. Whilst there are currently no computational models capable of reliably reproducing active-gaze sampling patterns in their entirety, there have now been a number of detailed empirical studies producing data and theoretical interpretations upon which a functional model could be based (^6,9,11,43^, for a review see^13^). There has been progress made capturing some aspects of visual sampling during driving-related tasks^49,76^, so there appears to be no reason that such a model could not be developed in the near future. The similarities in gaze patterns produced during Manual and Automation conditions in the present study should mean that gaze behaviours during automated driving can be modelled with only minor adjustments of model parameters. It is exciting to consider the potential of a unified model of gaze behaviour that would function irrespective of whether a human driver or automated vehicle is the controller. Not only could such a model allow the vehicle to interpret driver state prior to transitions of control (i.e. is the human ready to re-engage vehicle control?) but also classify driver intent regarding the purpose of their steering actions. It seems then that continuing to develop our understanding of the visual control of steering during manual driving will be crucial as advances in automation progress (cf.^8^, for a more in-depth discussion).

This potential does highlight questions around the degree to which the gaze pattern similarities observed in the present study will have applied relevance with respect to driver safety. It is clear that our highly controlled experimental conditions are not close in nature to typical real-world driving, and our findings of similar gaze behaviour between manual and automated driving modes should not be expected to generalize directly to naturalistic settings, where extraneous factors beyond monitoring the automated vehicle’s driving will often affect gaze behaviour (e.g. secondary tasks or interesting objects in the surrounding scene). We argue that the applied value of our findings is instead to tentatively establish an important baseline: *If the driver is doing nothing but monitoring the automated vehicle’s driving, then the driver’s gaze behaviour will be similar to what it would be if the driver was in manual control*. This statement is supported by our study; future studies can further test it in wider ranges of conditions, for example more challenging road geometries and driving scenarios. This baseline statement is important, because if it is true, then deviations between gaze behaviour exhibited by a driver supervising an automated vehicle and what would be expected from a manually controlling driver in the same situation can be taken to indicate that there are currently extraneous factors impacting the driver, beyond the monitoring of the automation. In turn, this suggests that such deviations could provide important diagnostic information for systems that monitor driver state and driver readiness to take over control.

It should be highlighted that although we show that some aspects of gaze behaviour are similar (e.g. waypoint tracking and gaze time headway) across driving modes, an important aspect of gaze behaviour that is not examined here is the extent to which gaze and steering is coordinated^8,77^. The simple road geometry and low steering demand of the current study did not elicit the large changes to gaze and steering that would be most useful for examining coordination, but steering and gaze coordination could have important consequences for manual control after takeover^8,77^. Future work should assess whether the similarities (and differences) between Manual and Automated driving can predict takeover success during transitions of control. Future work should also assess whether the gaze pattern similarities observed in the present study generalise to more complex road geometries and detailed visual scenes.

A final methodological note is to highlight the scope for developing further the mixture modelling method employed to decompose gaze into distinct groups. The principle of the mixture model was to separate a central guiding fixation mass (not tied to specific allocentric points in the world) from saccades to allocentric visual features that could serve as LAF candidates. This could scale up to any number of additional points, with points being added or removed based on the environment, task, and/or individual differences. There would appear to be great potential for employing such gaze models as an initial gaze processing step before gaze data are fed into models of steering control. The resultant probability assigned to each gaze fixation could then be used as an information weighting parameter that moderates the extent to which particular gaze fixations are fed into steering control, and in this way account for potential intermittency in the coupling between steering and gaze^13,30^.

## Conclusion

This study is the first to isolate the effects of active steering control on gaze behaviour. Gaze patterns were broadly similar across Manual and Automation driving modes when travelling around a simple oval track. The main change during Automation was the entire gaze pattern shifting further ahead (by around .2 s) with less time spent pursuing each waypoint tracking fixation. The similarities in gaze patterns across driving modes are consistent with the notion that an important determinant of gaze behaviour is stimulus motion, with gaze during manual driving moving closer due to the tighter perceptual-motor coordination required during active steering. The gaze pattern similarities are also consistent with the notion of executing a shared task across driving modes: observers monitoring vehicle motion. Under the shared task hypothesis, we propose that the gaze differences may have been due to (1) an increased emphasis on anticipation during automation, resulting in a slightly increased proportion of look-ahead fixations, and (2) an increased uncertainty about the automated vehicle’s state, resulting in additional sampling of preview information, as well as more frequent sampling of waypoints. The patterns were more pronounced when the trajectories of the automated vehicle differed from the trajectories generated by the participants themselves. Overall, these data suggest that models of gaze behaviour developed during manual driving tasks should generalise to capture gaze behaviours exhibited during automation, with the follow-on possibility that large differences in gaze behaviour during automation could mean that a driver is not closely monitoring the automated system.

## Methods

### Open science

Data collection was carried out in accordance with a pre-registration document submitted prior to the collection of these data^78^. However, after observing the data some analytic alterations were made to improve both the data quality and the focus and rigour of the manuscript. We improved the calibration procedures, considered only a subset of the track data, and developed a novel method of using mixture modelling to decompose gaze data into key components. The data and the software for running the experiment and analyses can be found on the Open Science Framework^79^.

### Participants

12 participants (7 females) were recruited from a University wide email list (mean age = 24.67 years, range = 19-33 yrs) and were reimbursed $\pounds 10$ for taking part. All participants had normal or corrected-to-normal vision, and a UK driving license (mean license months = 58, sd = 33.53). All participants gave written informed consent and the study was approved by the University of Leeds Research Ethics Committee (Ref: PSC-435), and complied with all guidelines set out in the declaration of Helsinki.

### Apparatus

The stimuli were generated using Vizard 5 (WorldViz, Santa Barbara, CA), a Python-based software for rendering perspective correct virtual environments, running on a desktop PC with Intel i7 3770. Participants sat on a fixed-based height-adjustable seat, with eye position 1.2 m high and 1 m from the display (field of view $89^{\circ } \times 58^{\circ }$). Display refresh and data recording was synchronized at 60 Hz. Participants steered using a force-feedback wheel (Logitech G27, Logitech, Fremont, CA). During the automated sections the steering wheel movement was matched to yaw rate in the scene. Eye-tracking was performed using Pupil labs version 1.7.130 (data format v1.13)^80^ on a laptop running linux (processer i5 8250U). The open source software was adapted to include in-house libraries that supported communication and synchronisation between the eye-tracking laptop and the experiment machine, and allowed for custom calibration procedures. Eye image data was collected binocularly at 60 Hz, at a resolution of $640 \times 480$, and a forward-facing camera recorded the scene at 30 Hz, at a resolution of $1920\times 1080$.

### Design and virtual environment

The experiment manipulated Driving mode: Manual (participant steered), Auto-Replay (replayed participant’s past steering), or Auto-Stock (replayed a pre-recorded stock trajectory). In a full dataset there were 16 Auto-Replay trials, 16 Auto-Stock trials, and at least 32 Manual trials. Only the manual trajectories that were replayed were used for inferences to ensure matched conditions could be compared.

**Figure 12:**
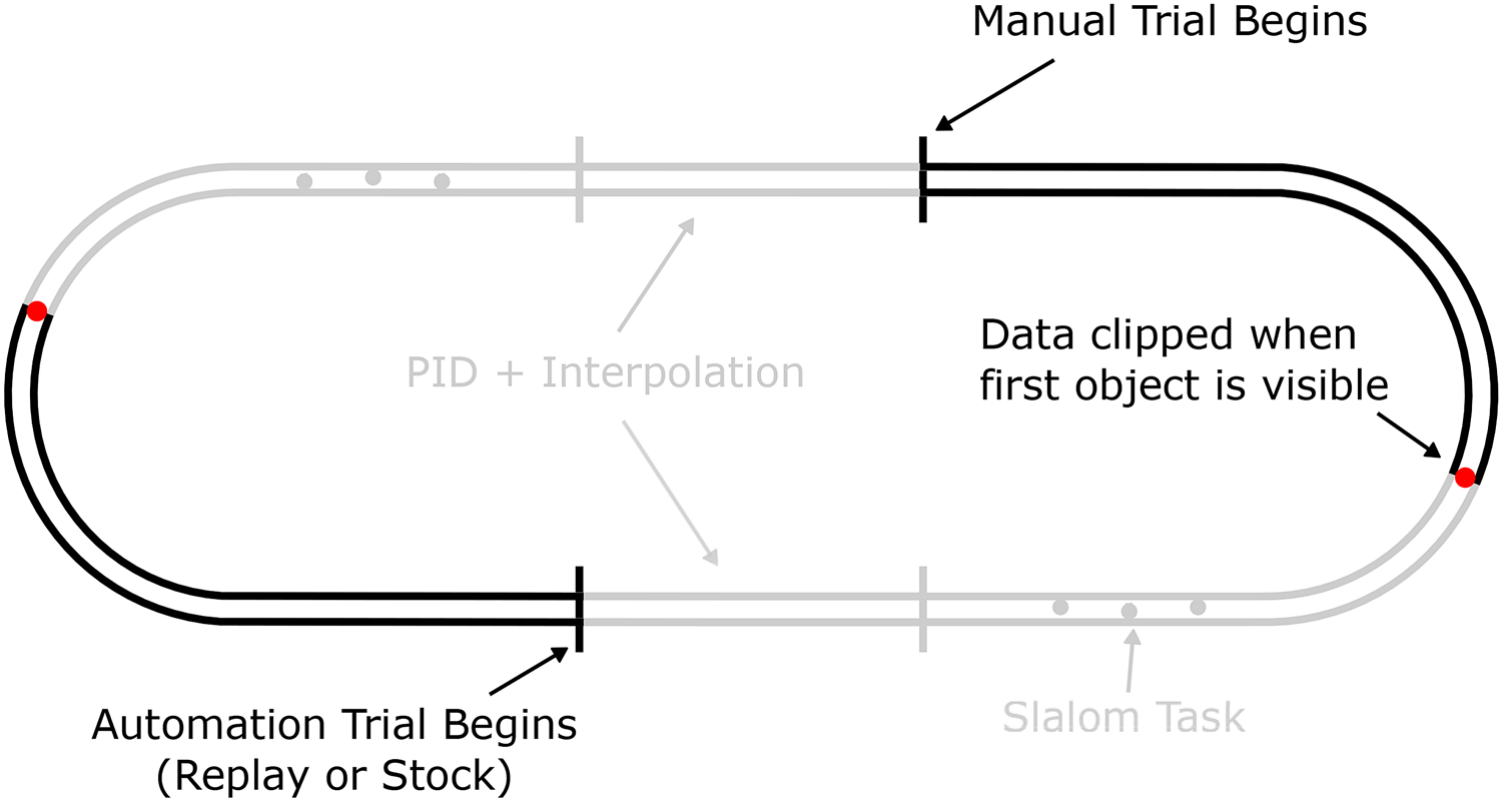
Annotated track. Driving was along a continuous track consisted of two 120 m straights and two bends of 25 m radius, with a road width of 3 m and at a speed of 8 m/s. The task alternated between Manual and Auto(mated) periods (Black). These periods were connected using an interpolation period that consisted of 4.5 s (36 m) of self-controlling PID control that smoothly brought the vehicle back to a central lane position, with a final .5 s (4 m) of linear interpolation of yaw, steering wheel, and lane position to ensure that the initial state precisely matched the beginning state of the next period. Gaze data were excluded from the frame where the first slalom object was on-screen (red). Sections of the track that were excluded from the analysis are greyed out.

The take-over point was consistent across successive laps (Fig. 12). Each period of manual driving was sequenced as follows: a 40 m straight section, a bend of 25 m radius, then a final 40 m straight section with three objects placed in various slalom configurations. For reasons of brevity, the slalom components will be considered in a separate manuscript. The automation period consisted of three sections: (i) a first 40 m interpolation period, (ii) a playback period of equal length to the manual period, and (iii) a final 40 m interpolation period. The interpolation periods smoothly brought the vehicle from the end-state of the previous (manual or auto) period to the desired initial state (of yaw, steering wheel, and lane position) of the next (manual or auto) period. The playback period replayed either a stock trajectory (Auto-Stock) or one of the participant’s previous trajectories (Auto-Replay). A single beep (490 Hz; 0.4 s) signalled a transfer from Automated to Manual driving; a double beep (590 Hz; 0.1 s each with a 0.5 s delay) signalled a transfer from Manual to Automated. Transfer of control was immediate. A single letter (‘A’ for Automated; ‘M’ for Manual) was placed on the far left of the screen to denote the current driving mode.

### Procedure

Participants experienced eight practice laps of the track to become familiar with the simulator dynamics and the task instructions. The first four practice laps were entirely under manual control; then over the final four laps four handovers of control were presented: two handovers from automation to manual control and two handovers from manual to automation control.

The experiment proper was conducted over four blocks that each consisted of eight minutes of driving and four minutes of eye-tracker calibration, with the opportunity for a break in between each block to mitigate against driver fatigue. Each block began with four manual control laps of the track. The driver then alternated between a trial of manual and a trial of automated driving (mean trial length = 19.78 s, sd = .084s). The sequence of Auto-Replay and Auto-Stock driving modes was randomized, as was the order in which the Manual trials were replayed.

The intention was that participants believed the Auto-replay and Auto-stock trials were generated by a separate automated vehicle controller. To avoid participants easily recognising their own trials (with associated distraction or potential bias), Manual trials that had potentially identifiable characteristics (e.g. large errors or failures to adhere to the steering instructions) were excluded from Replay. Smaller idiosyncrasies (such as jerky steering) were not removed. Interleaving the Replay and Stock trials, and replaying trials in a different order to which the trials were initially generated appeared to avoid detection of the method of automation. While participants sometimes reported that the system was learning from their behaviour, no participant identified that the automated behaviour was a replay of their own steering. When asked “How human-like did you find the automated steering control” the mean rating was 59.38%. Further, when asked “How similar to your own steering behaviour did you feel the automated vehicle was?” the mean rating was 61.11%.

To ensure that the trajectories taken during Manual steering were as “natural” as possible (i.e. not unduly constrained by task instructions) drivers were simply told to “steer within the road-edges” when actively steering. In previous research similar instructions to stay within lane have been shown to produce naturalistic steering behaviours using controlled simulated scenes similar to those used in the present displays (e.g.^32^). For the Automated task participants were instructed to “Keep their hands on the wheel as if steering, ready to take-over”.

### Calibration

During the experiment gaze measures were repeatedly calibrated at the beginning and end of each block using a 3 by 5 grid spanning the screen area where the road is most of the time. The repeated measurements ensured that calibration drift could be detected. Before analysis the gaze data were adjusted offline using the estimated marker positions recorded at the beginning and end of each block, then weighted according to distance in time from each recorded calibration.

The overall mean calibration error (calibration compared to the calibration data itself) was 0.8 degrees and overall mean verification error (calibration compared to data from other end of the block) was 2.1 degrees. Due to the weighting scheme the typical mean error is estimated to be approximately 1.5 degrees.

### Estimating a gaze time headway signal

To estimate how far ahead a driver is looking at a specific time, the gaze data (from the eye image cameras) must be integrated with the corresponding perspective-correct view in the virtual environment (given by the vehicle position, orientation, and road geometry). There are two steps to this process.

First, the gaze data is projected to screen coordinates, which acts as the common coordinate system for the gaze data and the virtual environment. For each video frame, the screen coordinates of the optical markers (see markers in Fig. 2) define the transformation matrix that projects gaze from the video frame to the screen coordinates. Since the forward-facing scene camera is fixed on the participant’s head, the projection effectively controls for variability in head position between frames. This method results in horizontal and vertical gaze positions in screen coordinates at 60 Hz for each frame of the rendered scene image.

Secondly, from screen coordinates gaze can be projected into the virtual world. However, the screen-to-world projection should be treated cautiously. Projective geometry means that distance ahead tends towards infinity as the vertical angle tends towards zero (i.e. in line with the horizon), meaning that small changes in gaze (e.g. due to noise or miscalibration) can result in very large differences in time headway when projected through to the world, and this issue could skew measures of central tendency (illustrated in Supporting Information). This issue is particularly problematic when a driver is looking close to the horizon (often the case during bend approach; see Fig. 2), since a relatively minor miscalibration could result in gaze being projected far beyond the road when the driver was looking at the road (leading to spurious, large time headway estimates).

To control for estimating disproportionately large gaze time headway values, the assumption was made that gaze will mostly be directed towards the road ahead rather than to the region between the road and the horizon. Indeed, 90% of reliable gaze estimates fall within 5 degrees from the midline. Since the primary focus of this manuscript is to estimate with precision *how far ahead* on the road drivers are looking (rather than the precise spatial location), projection errors are avoided by first mapping the screen coordinates of gaze to the closest point (in screen-coordinates) of the track midline (see Fig. 2). The closest point along the track midline is called the *gaze midline reference*. Gaze time headway is the distance *along the midline* between the closest point to the midline from the vehicle’s current position and the gaze midline reference point (converted into seconds). This method ignores variations in the vehicle’s lane position, but the contribution of lane position changes to time headway is small compared to the potential influence of projection error. To avoid erroneously including gaze data when participants were not looking at the road (e.g. gaze directed at the visible markers or off-screen), gaze data were excluded when: 1) the pupil-detection confidence rating was below 0.6, 2) gaze was estimated to land outside the marker surface, 3) gaze was greater than 20 degrees away, in any direction, from the midline reference, or 4) gaze was estimated to be looking at any of the reference markers. These four exclusion criteria removed a combined total of 8.4% of the gaze data.

## Supplementary information

Supplementary Information
