# Supplementary material for: Drivers use active gaze to monitor waypoints during automated driving
Source: Sci Rep. 2021 Jan 8;11:263. doi: 10.1038/s41598-020-80126-2 (PMC7794576; doi:10.1038/s41598-020-80126-2)
Supplement: Supplementary file 1 — Supplementary Information [file 41598_2020_80126_MOESM1_ESM.pdf]

Supplementary Information: Drivers use active gaze to monitor waypoints during automated driving

Callum Mole, Jami Pekkanen, William E. A. Sheppard, Gustav Markkula, and Richard M. Wilkie

This document lists supporting text, tables, and figures, in that order.

SI Appendix 1. Mixture Model Details

Described here are the mathematical specifications of the mixture model, and the techniques for fitting and sampling from the fitted models.

Each gaze fixation (y_i) is considered to have an associated (but unobserved) weight vector, $(w_{i,1},\ldots,w_{i,K})$, that specifies the probability of belong to each regression model (k). Each regression model has parameters θ_k and a mixing proportion ω_k (i.e. the cluster weight). ω_k is the average of the normalised weights for each model ($\omega_k = \frac{1}{n}\sum_{i=1}^{n} w_{i,k}$). The time headway probability density is the sum of the weighted mixture regression model densities:

$$p(y|\theta,\omega) = \sum_{k=1}^{K} p(y|\theta_k)\omega_k$$

Each linear regression model is specified with parameters $\theta_k = \begin{bmatrix}\alpha_k & \beta_k & \sigma_k\end{bmatrix}$:

$$y_i = \alpha_k + \beta_k x + \varepsilon_i \qquad \varepsilon_i \sim N(0,\sigma_k)$$

The first regression model is the primary GF model (θ_G), with a fixed slope (β) of zero but freely varying intercept (α) and standard deviation (σ). Any additional regression model for a point of special interest has a fixed slope of -1 (Manuscript Figure 4D). Since the fitting process is susceptible to outliers and gaze data is generally noisy we also add a noise cluster (θ_N) to pick up outlying data that cannot be confidently associated with the main gaze clusters. The noise cluster has fixed parameters: a slope of zero, intercept fixed to the grand mean ($\bar{x}$) of the data, and a standard deviation of twice the grand standard deviation ($\bar{\sigma}$) of the data. If one specifies the number of salient points as S, the parameters are as follows:

$$\begin{bmatrix}\theta_G\\ \theta_1\\ \vdots\\ \theta_S\\ \theta_N\end{bmatrix} = \begin{bmatrix}\alpha_G & 0 & \sigma_G\\ \alpha_1 & -1 & \sigma_1\\ \vdots & \vdots & \vdots\\ \alpha_S & -1 & \sigma_S\\ \bar{x} & 0 & 2\bar{\sigma}\end{bmatrix}$$

The model is fitted using the Expectation-Maximisation algorithm [1]. The steps are as follows. If the component parameters (θ_k) are known, then calculating the mixture proportions (ω_k) is relatively simple. If we take n as the number of observations, from calculating the log-likelihood of the mixture model:

$$\log p(y|\theta,\omega) = \sum_{i=1}^{n}\log\sum_{k=1}^{K} p(y_i|\theta_k)\omega_k$$

One gets the normalised weights for each observation, $(w_{i1},\ldots,w_{iK})$. This is the *Expectation* step. Once these weights are known the weighted averages and standard deviations that *maximise* the likelihood of the data, given the current weights (*expectation*) can be computed. By iterating from *estimating weights* to *estimating parameters* we can increase the likelihood on every step.

If appropriate initial values are set the algorithm converges to the posterior mode. The current implementation of the model had two gaze clusters (the GF cluster bend entry clusters) and a fixed noise cluster, therefore four free parameters

$(\beta_G, \beta_E, \sigma_G, \sigma_E)$. Since the final fit is sensitive to initial values we adopt a sparse grid of initial values and select the fit with the highest likelihood. The initial value of β_G was fixed at $\bar{x}$. The initial values for $\beta_E, \sigma_G, \sigma_E$ were gridded around the intercept corresponding to the point of highest gaze density in the pooled dataset (for β_E) and $\bar{\sigma}$ (for σ_G and σ_E). The gridding process resulted in 27 separate fits.

To retain the trial-by-trial structure when sampling from the model, each trial time headway density is estimated using the regression weights, $(w_{i1}, \ldots, w_{iK})$, for each gaze observation for that trial. These trial densities are then averaged across trials to get a participant's average time headway density. From these average densities the mean, standard deviation, and cluster weights are calculated. Individual fits for every condition can be viewed in supplementary materials here. The mixture model implementation can be found in an online repository [2].

SI Appendix 2. Bayes Model Details

This appendix describes the details of the distributional models used for inference. For all the models used in this paper, each participant has a single observation per condition (a median or a mean). In all cases, the spread of participant means are approximated by a normal distribution. We wish to model one factor (Driving Mode) with three levels (Manual, Auto-Replay, Auto-Stock). Treating Manual as the reference condition (i.e. the model's intercept; β_M) means that the coefficients for Auto-Replay (β_R) and Auto-Stock (β_S) are modelled as deflections from the Manual condition, controlled by binary variables (R, S) that denote the presence of the condition.

Weakly informative priors are specified based on previous literature (the inferences remained unchanged when we tested a range of priors). The distributional model for all time headway inferences is of the form:

$$
\begin{aligned}
y_i &\sim Normal(\mu, \sigma) \\
\mu &= \beta_M + \beta_R R_i + \beta_S S_i \\
\beta_M &\sim Normal(2.5, 2) \\
\beta_R &\sim Normal(0, 1) \\
\beta_S &\sim Normal(0, 1) \\
\sigma &\sim Cauchy(0, 1)
\end{aligned}
$$

The tracking duration model was fitted with a log normal distribution, with the priors given below:

$$
\begin{aligned}
y_i &\sim Lognormal(\mu, \sigma) \\
\mu &= \beta_M + \beta_R R_i + \beta_S S_i \\
\beta_M &\sim Normal(0, 1) \\
\beta_R &\sim Normal(0, 1) \\
\beta_S &\sim Normal(0, 1) \\
\sigma &\sim Cauchy(0, 1)
\end{aligned}
$$

All models were fitted in R using the package brms [3], using 1200 iterations split into four chains.

References

1. Moon, T. K. The expectation-maximization algorithm. *IEEE Signal processing magazine* **13**, 47–60 (1996).
2. Mole, C. *et al.* Gaze behaviour in automation (2020). Retrieved from osf.io/f2n4c.
3. Bürkner, P.-C. *et al.* brms: An r package for bayesian multilevel models using stan. *J. statistical software* **80**, 1–28 (2017).

ID	Manual	Auto-Replay	Auto-Stock
1	.018	.15	.11
2	.019	.019	.028
3	.002	.085	.032
4	.062	.18	.038
5	.004	.000	.000
6	.000	.075	.076
7	.2	.19	.14
8	.03	.15	.1
9	.006	.096	.18
10	.12	.18	.38
11	.015	.000	.089
Mean	.04	.10	.11
SD	.06	.07	.11

SI Table 1. Amount of gaze probability assigned to the noise cluster for each driving mode (1 = 100%).

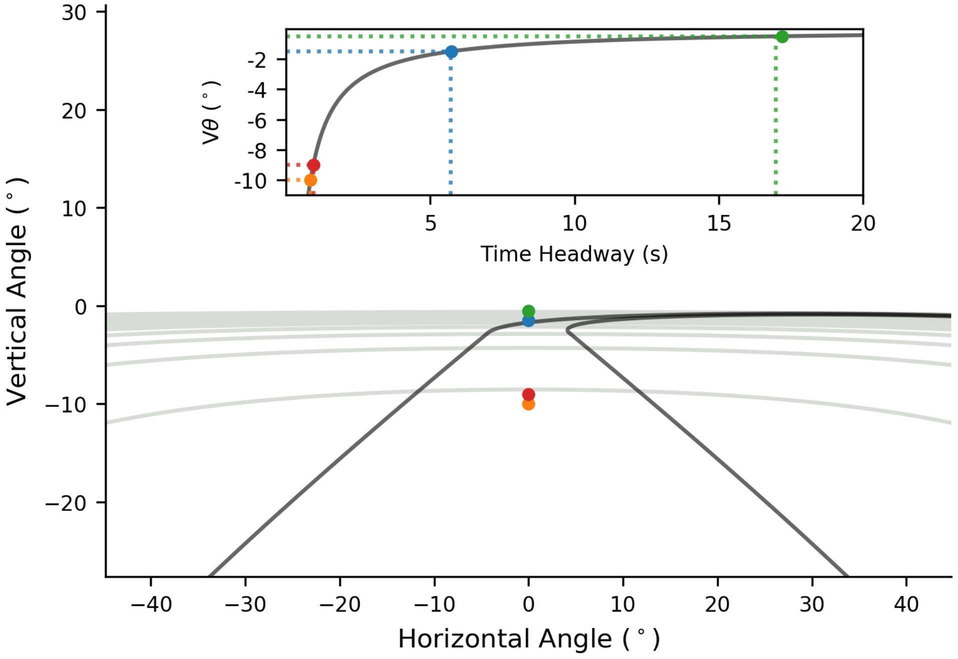

SI Fig 1. Projection Error. A perspective view of the road showing paired near and far hypothetical gaze points, offset by one vertical degree. Though optically one vertical degree is a small difference, when projected through the world even small optical differences can result in very different conclusions of where gaze lands. The inset shows the exponential relationship of vertical angle to time headway, the closer gaze is placed to the horizon. When gaze is placed near, there one vertical degree causes little difference in gaze time headway (orange = .85 s; red = .95). When gaze is placed far, however, one vertical degree causes a large difference in time headway (blue = 5.7 s; green = 17.2 s). To further illustrate, grey contours are placed at successive 1 s gaze time headways.

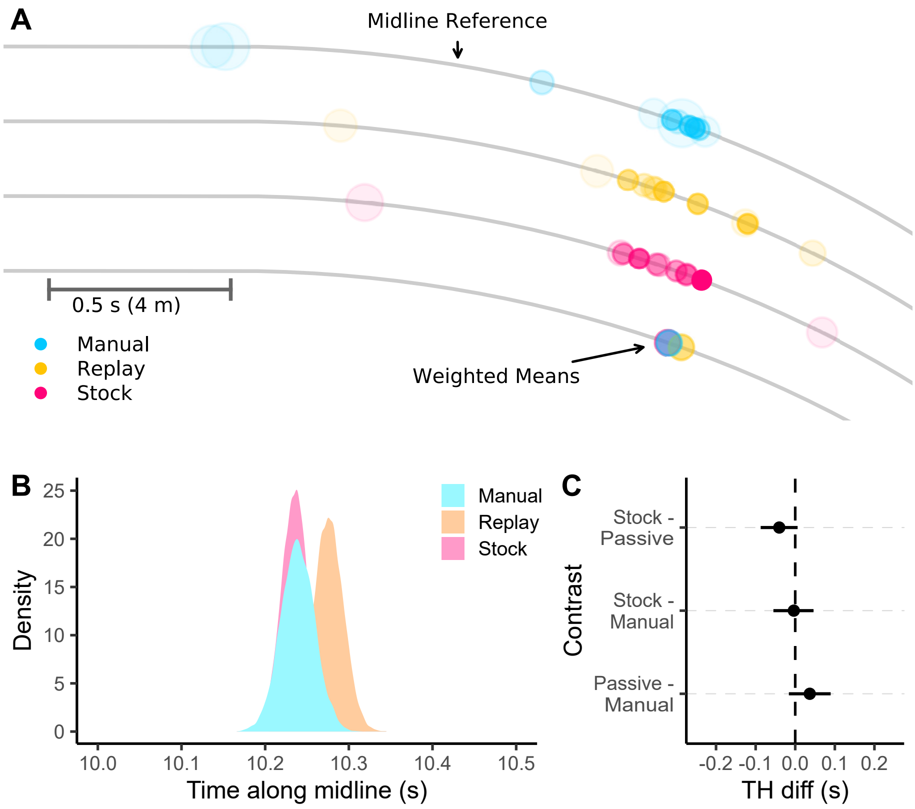

SI Fig 2. Entry Fixation Placements. After model fitting, for each participant the Entry Fixation (EF) cluster has an intercept, in units of time along the midline, and a standard deviation. Upon inspection of the individual fits it seems that participants with a high EF cluster standard deviation often did not clearly fixate on a single point (e.g. participant 5, SI Fig 8). For these participants, the EF categorisation did well at separating gaze which diverged from the primary GF cluster, but did poorly at estimating a single point of fixation. Therefore, when estimating where along the track participants looked, on average, each observation (intercept) is weighted by its precision ($\frac{1}{\sigma^2}$) to avoid poor fits biasing the population estimate. A) The Entry fixation placement for each participant, for each driving mode, placed along the midline reference. The precision of each EF cluster is indicated by the dot size and transparency (highly certain observations are small and bold). The weighted means are also shown. B) Bayesian weighted linear regression was performed, with the posterior estimates of the mean intercepts for each condition shown. C) The posterior contrasts between each driving mode condition. Any differences are estimated to be very small (<.09 s, or < 71 cm), so we recommend that the EF fixation placement be considered practically equivalent across conditions.

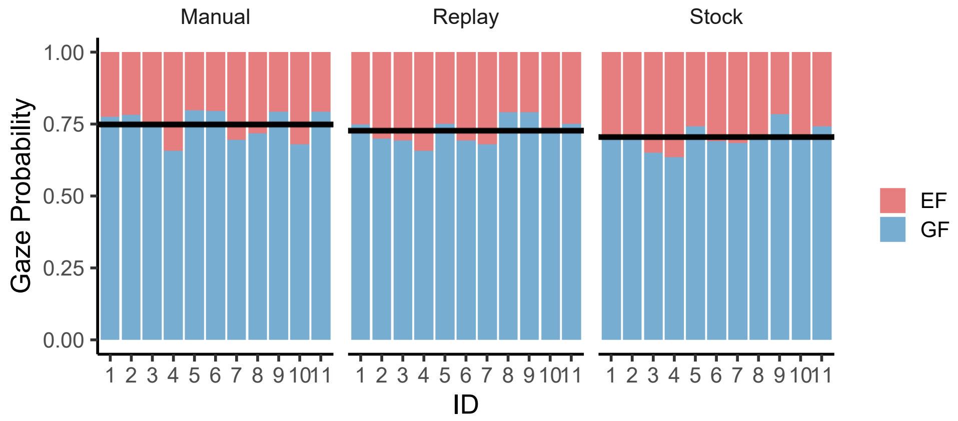

SI Fig 3. Fitted Cluster Weights. The fitted cluster weights across driving modes, for each participant. For each participant the gaze probability associated with the noise cluster has been removed (see Table 1), with the remaining gaze probability normalised across entry fixation and guiding fixation clusters. The black lines denote the driving mode mean.

Individual Fits

Listed below are the mixture modelling individual fits, for each driving mode, entitled with the participant number and the driving mode. The caption for all figures is as follows: *A) Gaze data, with gaze time headway (TH) along the ordinate and Time into Trial along the abscissa. Gaze fixation data are shaded according to the probabilities of belonging to Guiding Fixations (GF; blue), Entry Fixations (EF; red) or Noise (Grey) clusters. B) The regression lines and standard deviations of each model, from which a weighted sample is taken. C) Smoothed average cluster weights across the track. D) Raw gaze TH density (solid line) overlaid with the fitted gaze TH density (dashed line). E) The fitted gaze TH density decomposed into mixtures of GF (blue) and EF (red).*

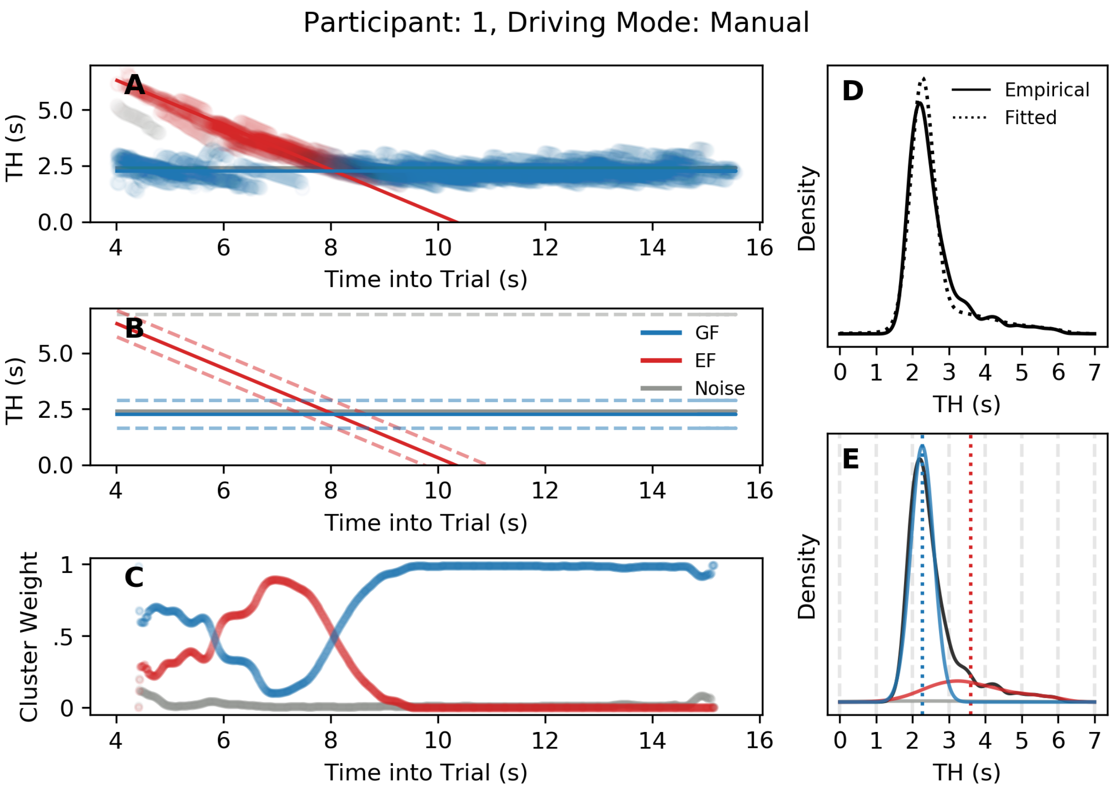

SI Fig 4

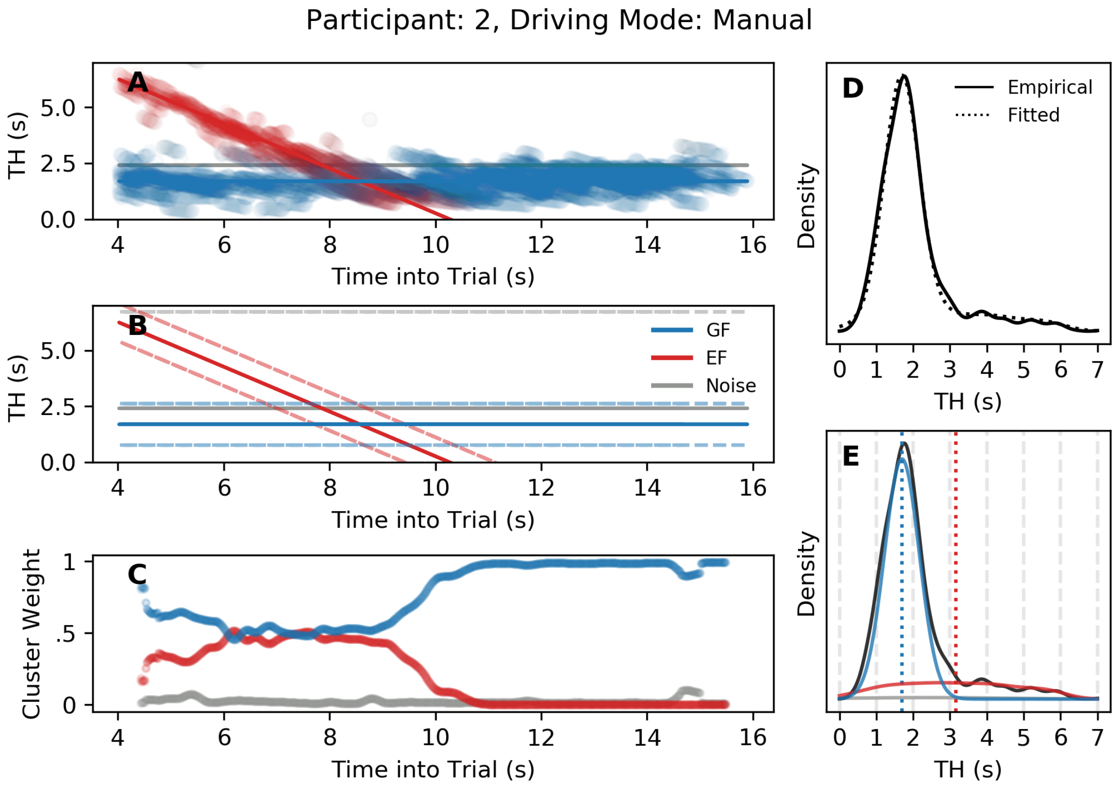

SI Fig 5

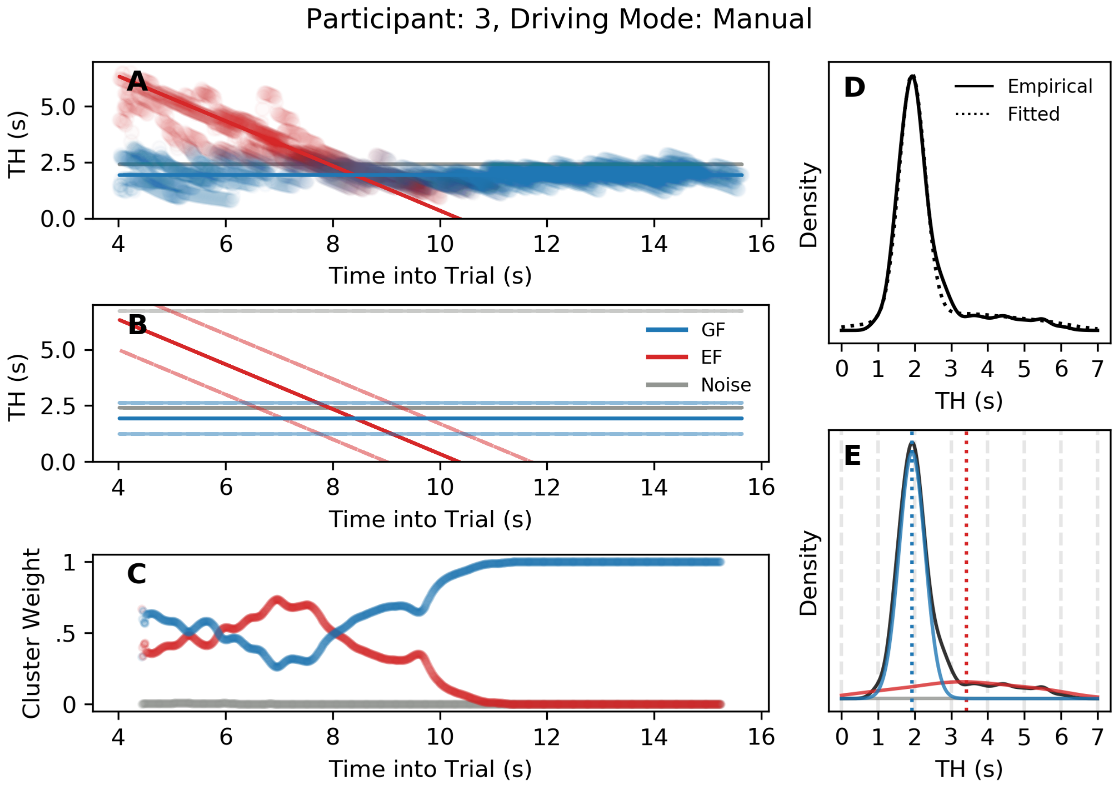

SI Fig 6

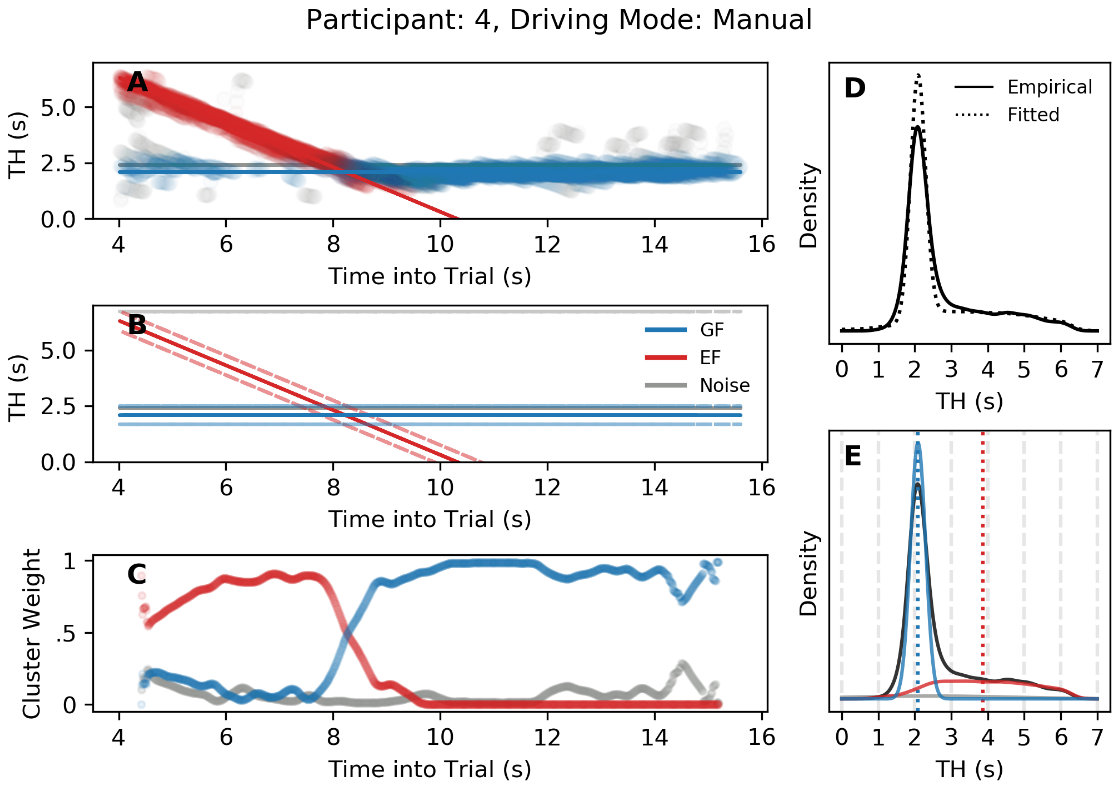

SI Fig 7

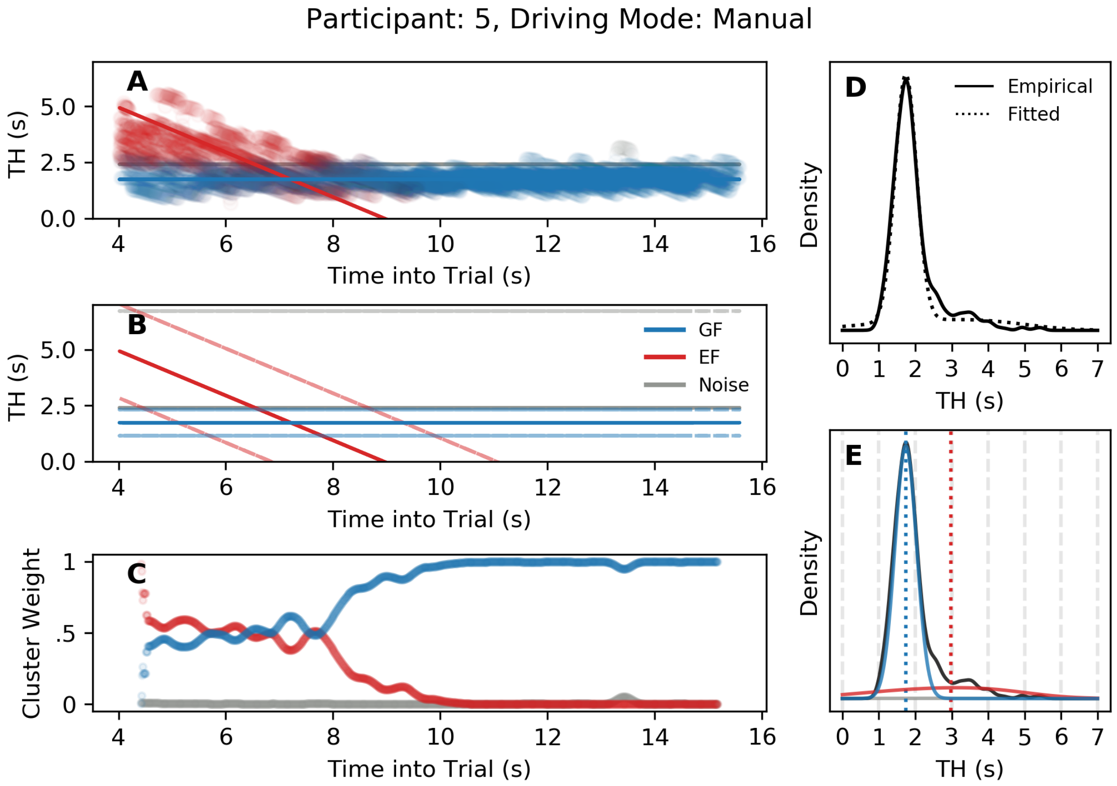

SI Fig 8

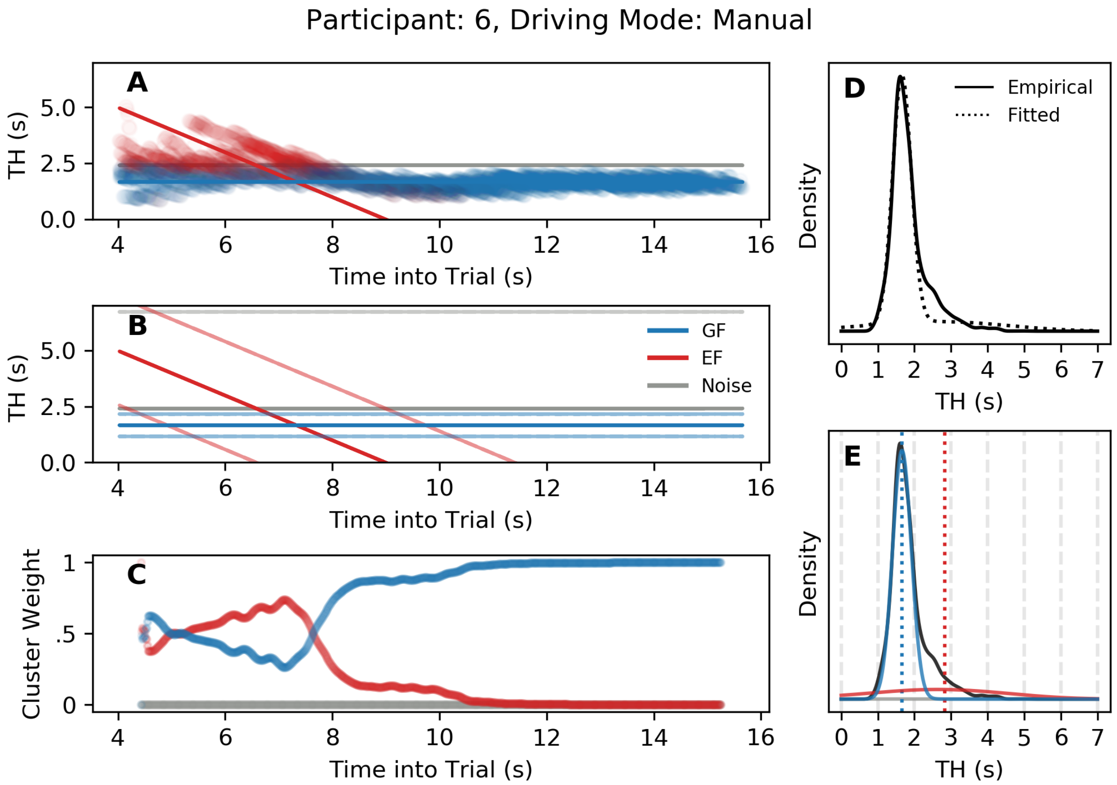

SI Fig 9

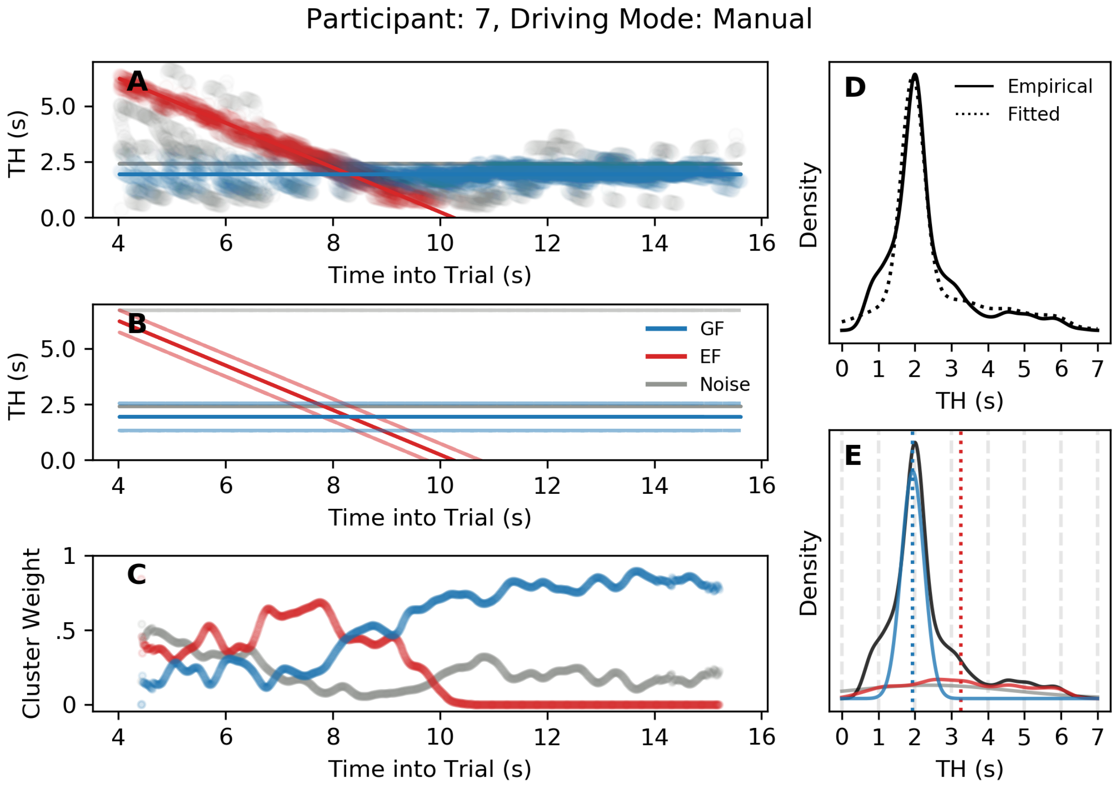

SI Fig 10

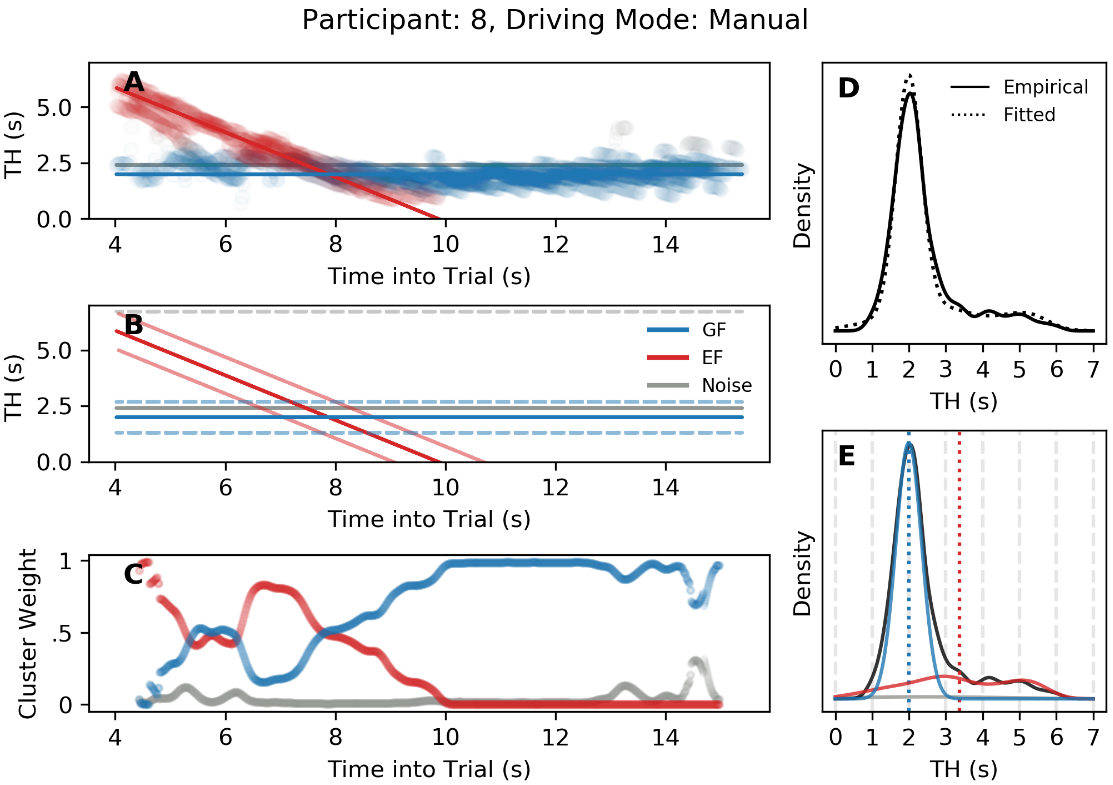

SI Fig 11

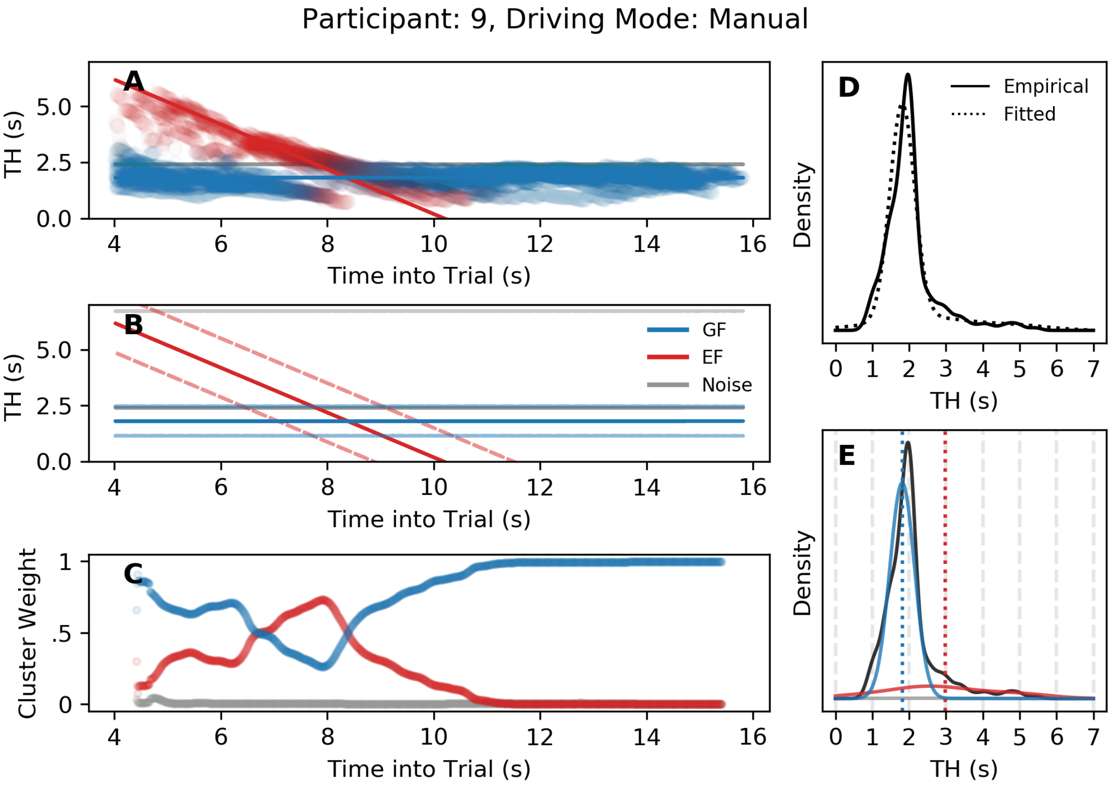

SI Fig 12

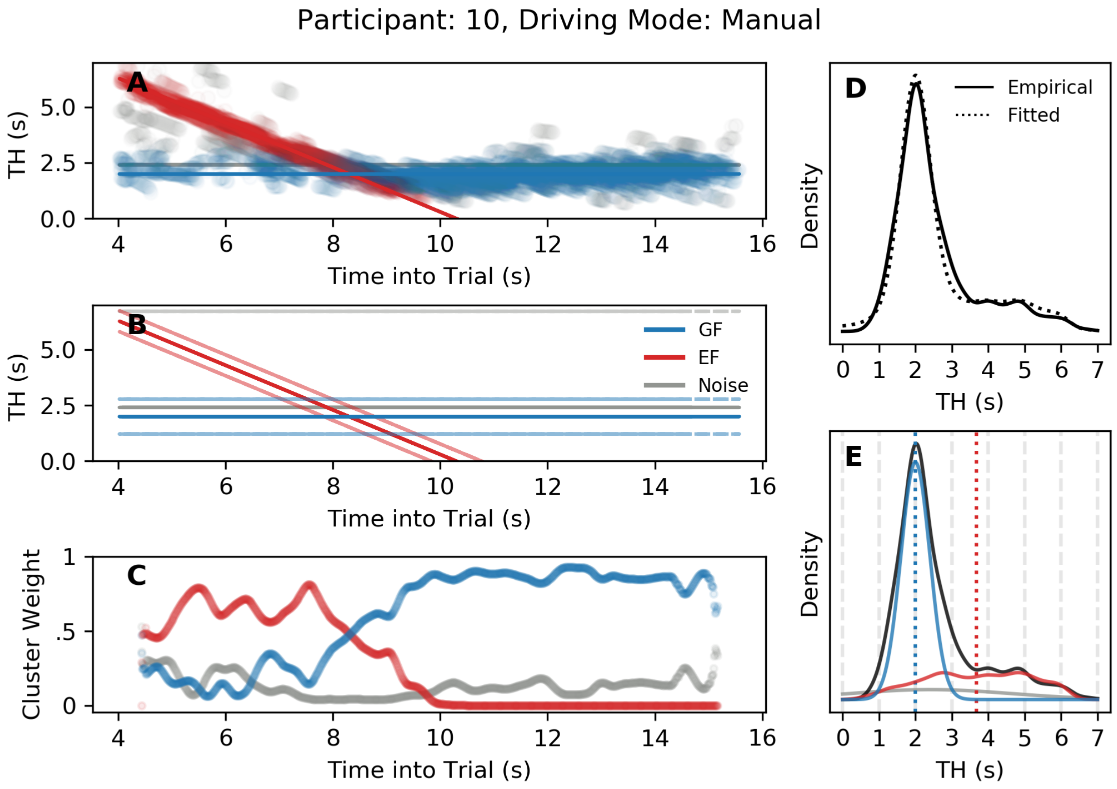

SI Fig 13

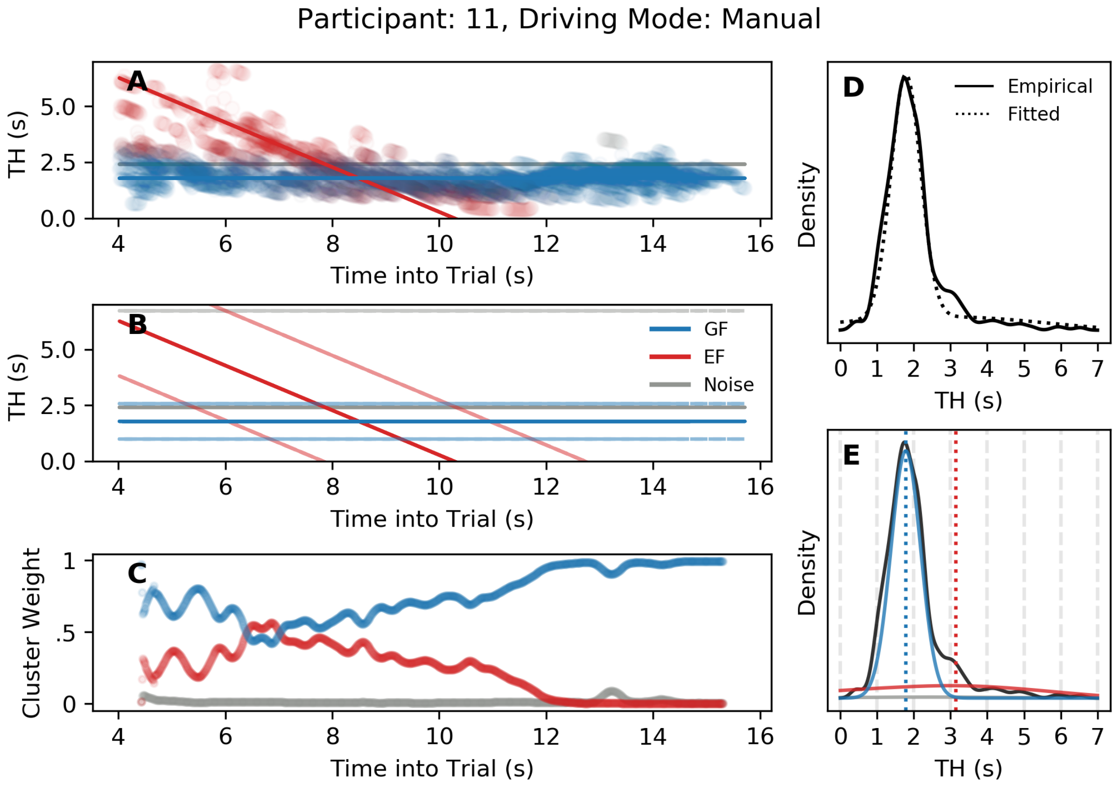

SI Fig 14

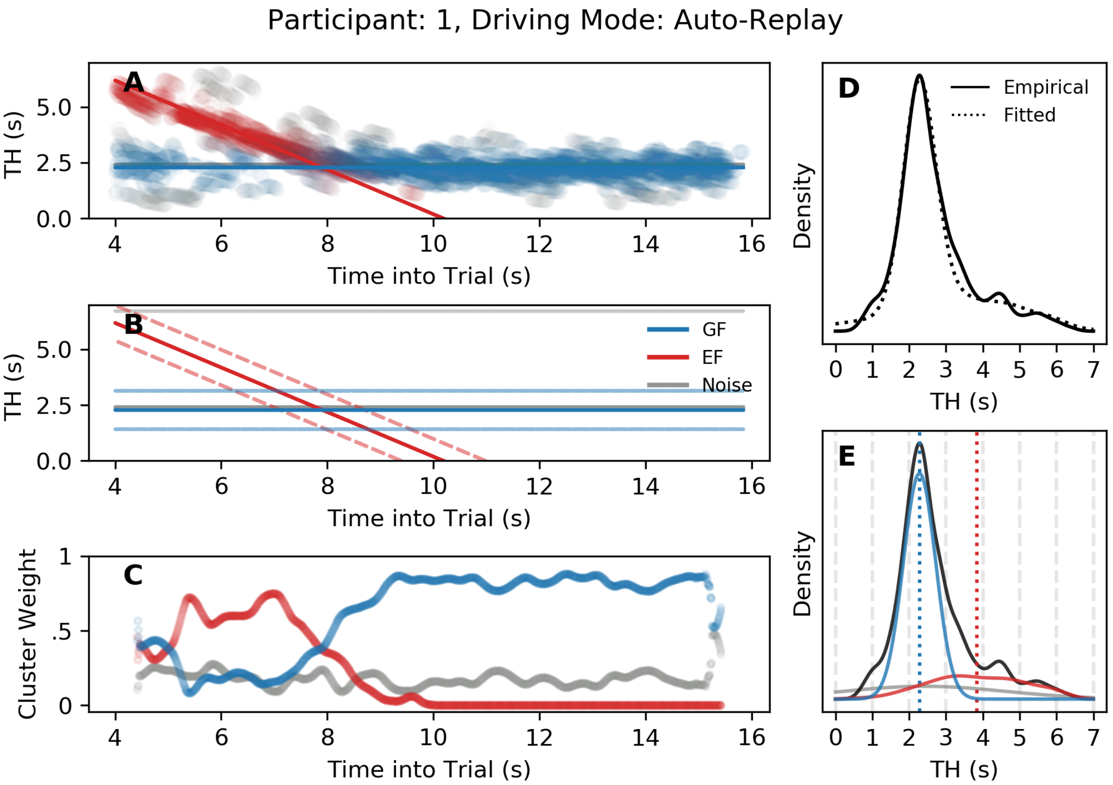

SI Fig 15

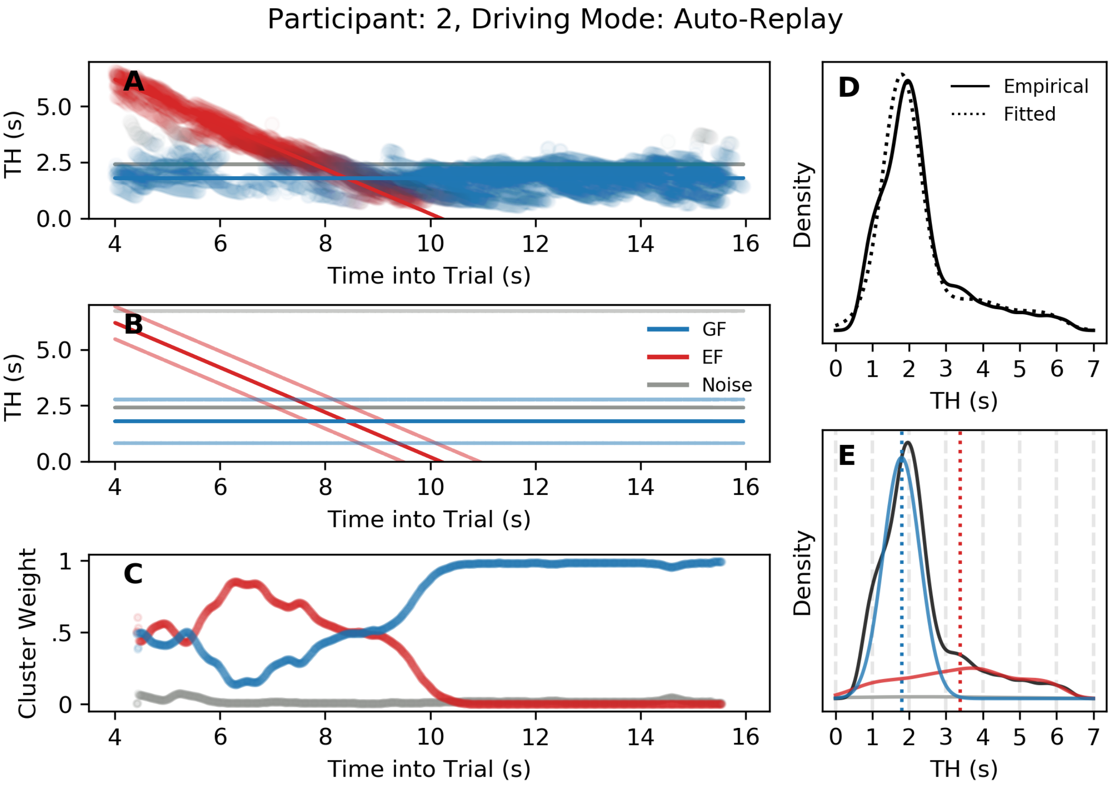

SI Fig 16

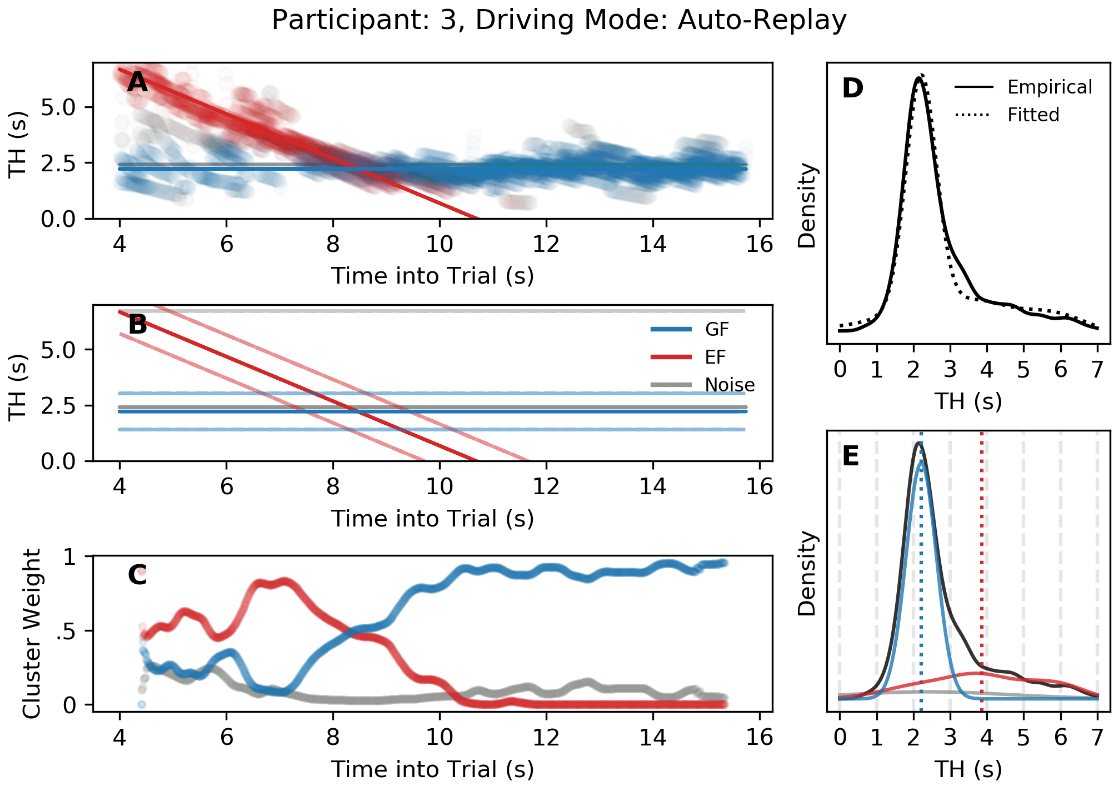

SI Fig 17

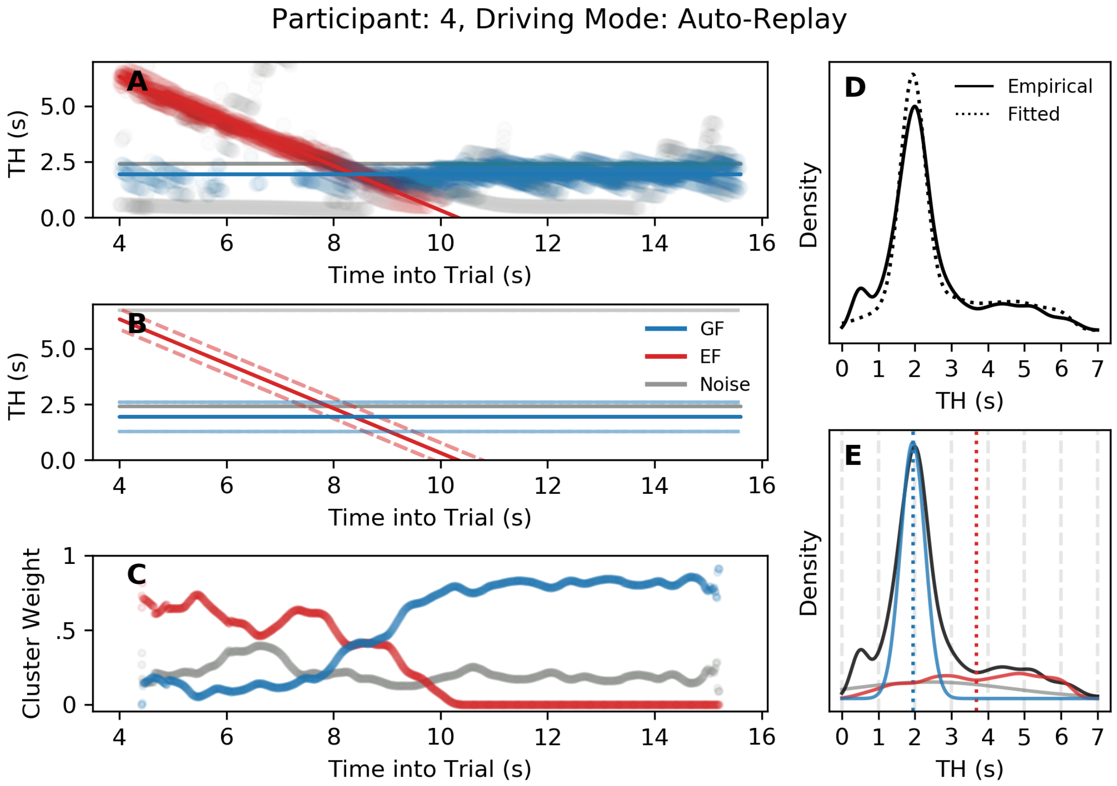

SI Fig 18

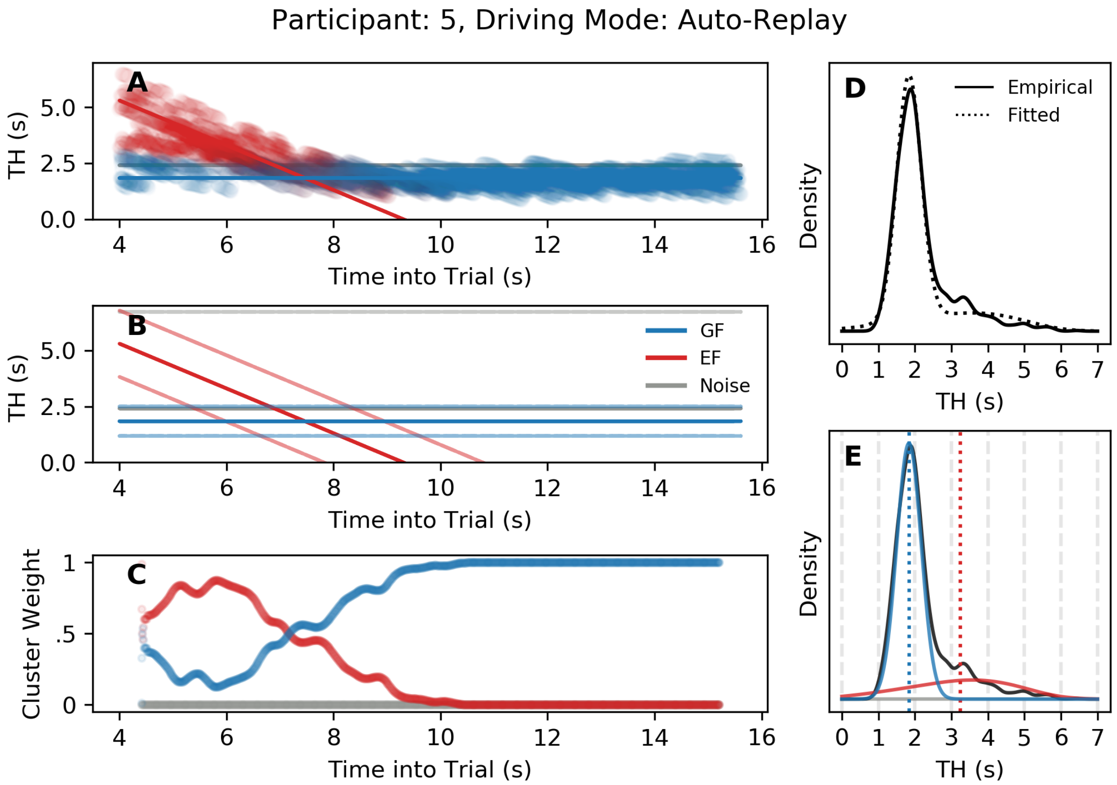

SI Fig 19

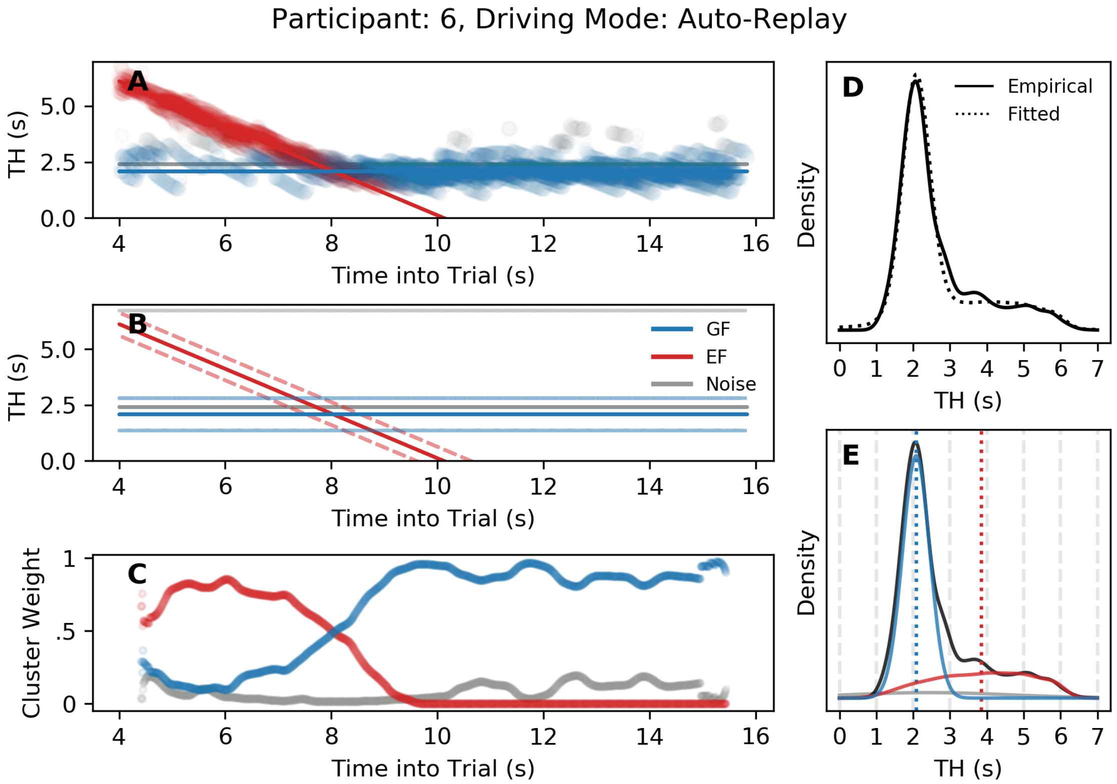

SI Fig 20

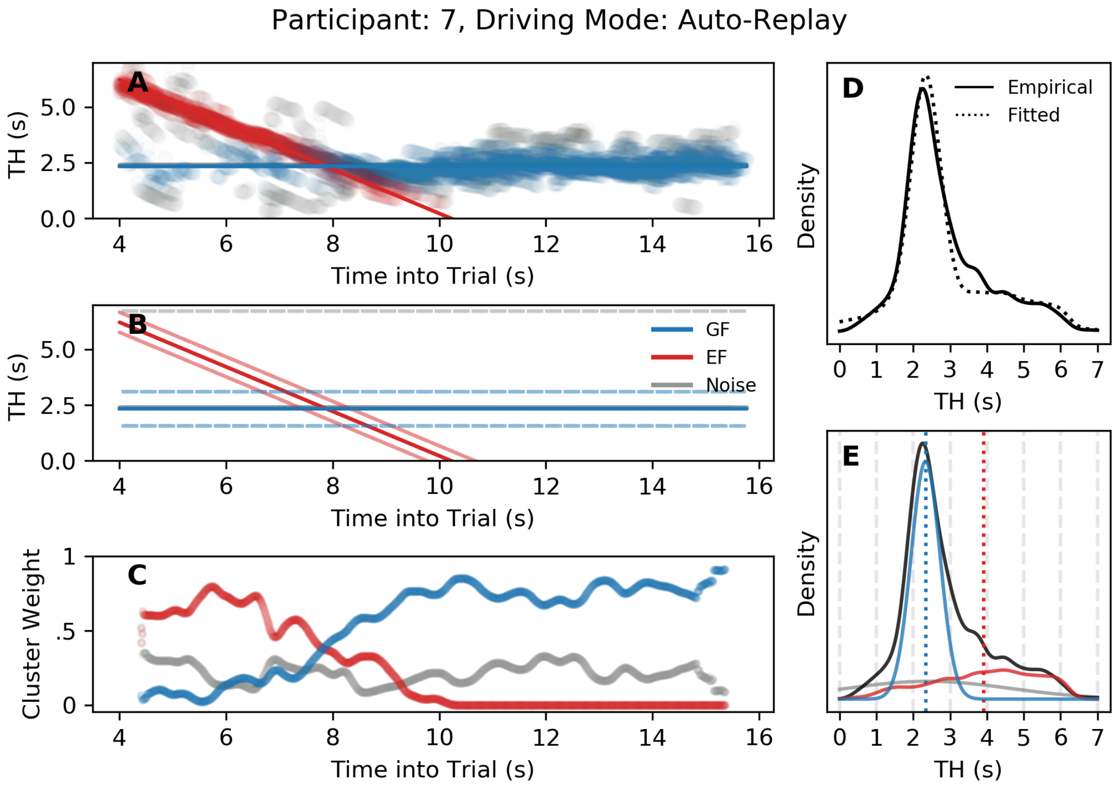

SI Fig 21

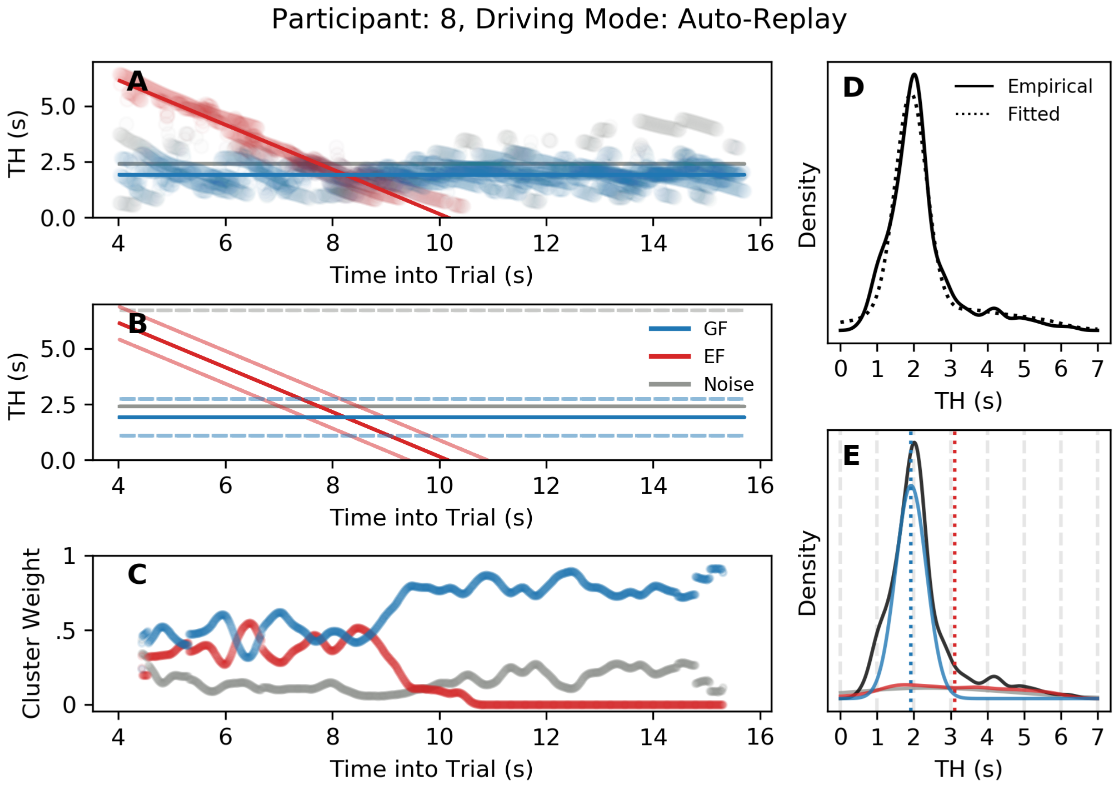

SI Fig 22

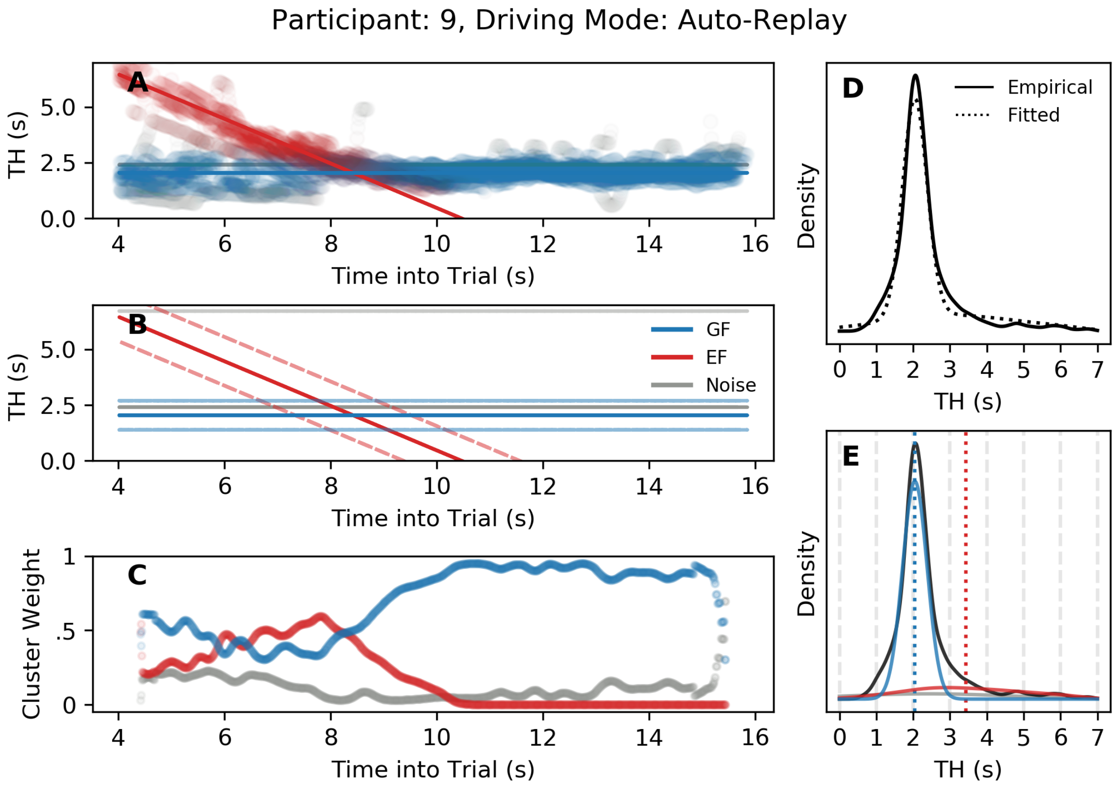

SI Fig 23

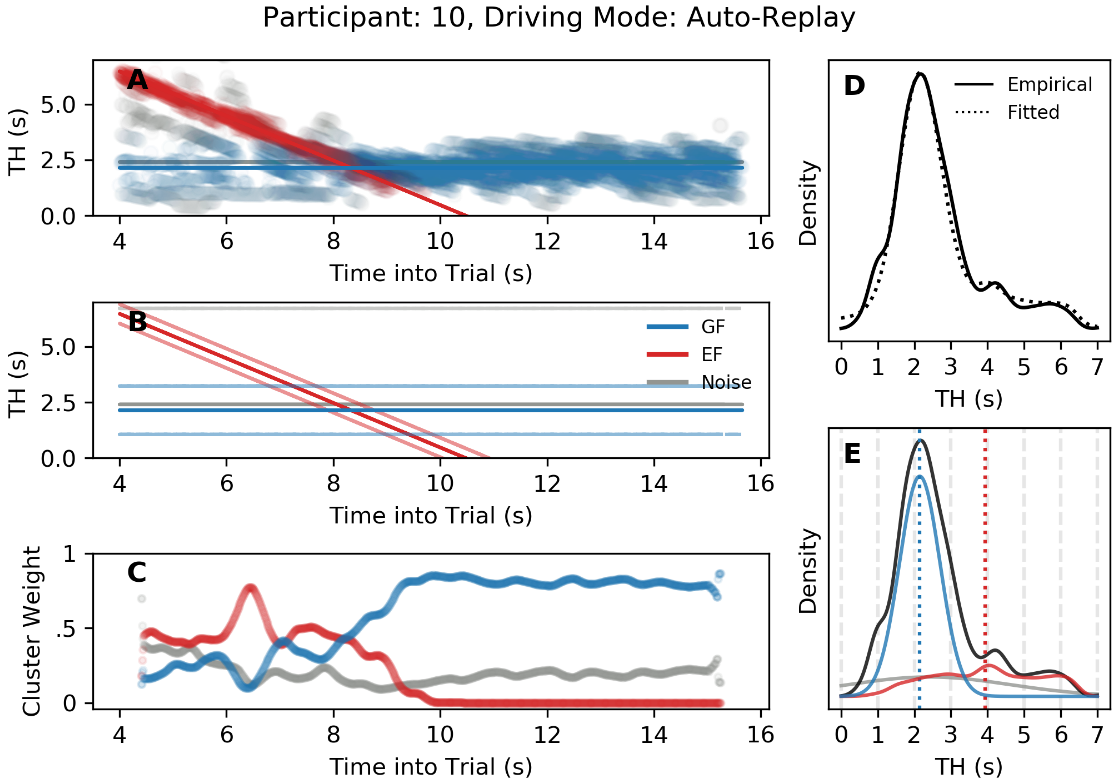

SI Fig 24

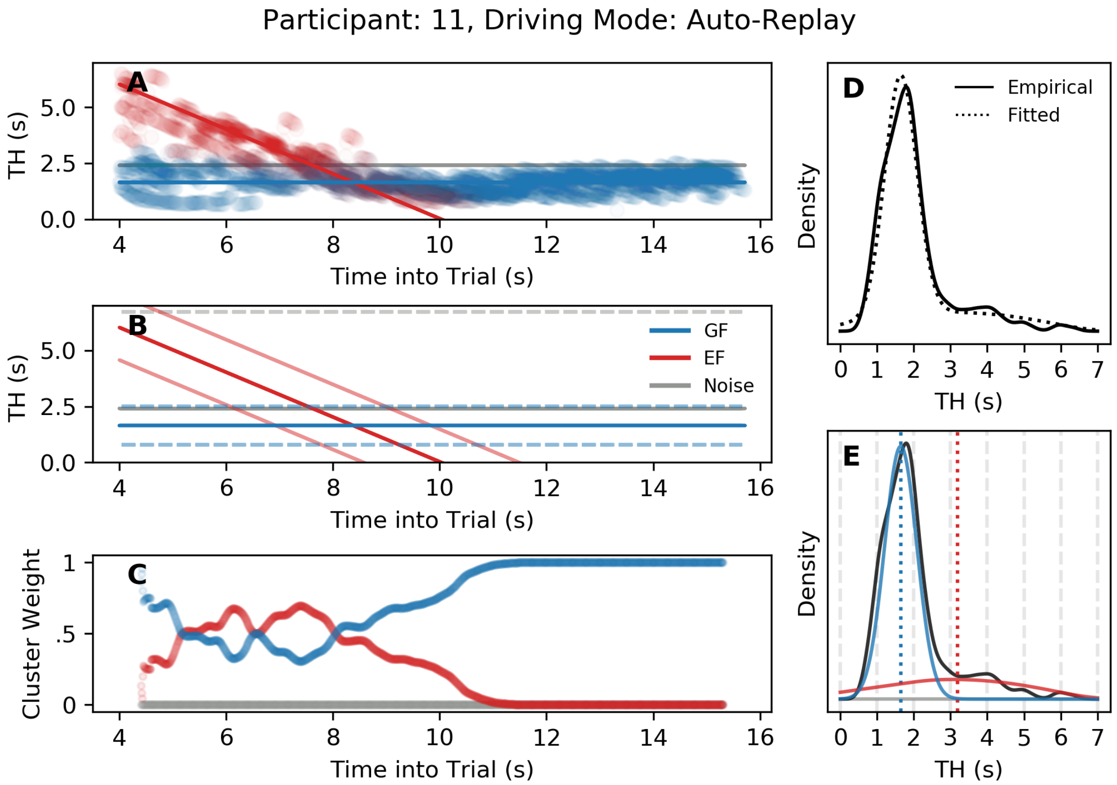

SI Fig 25

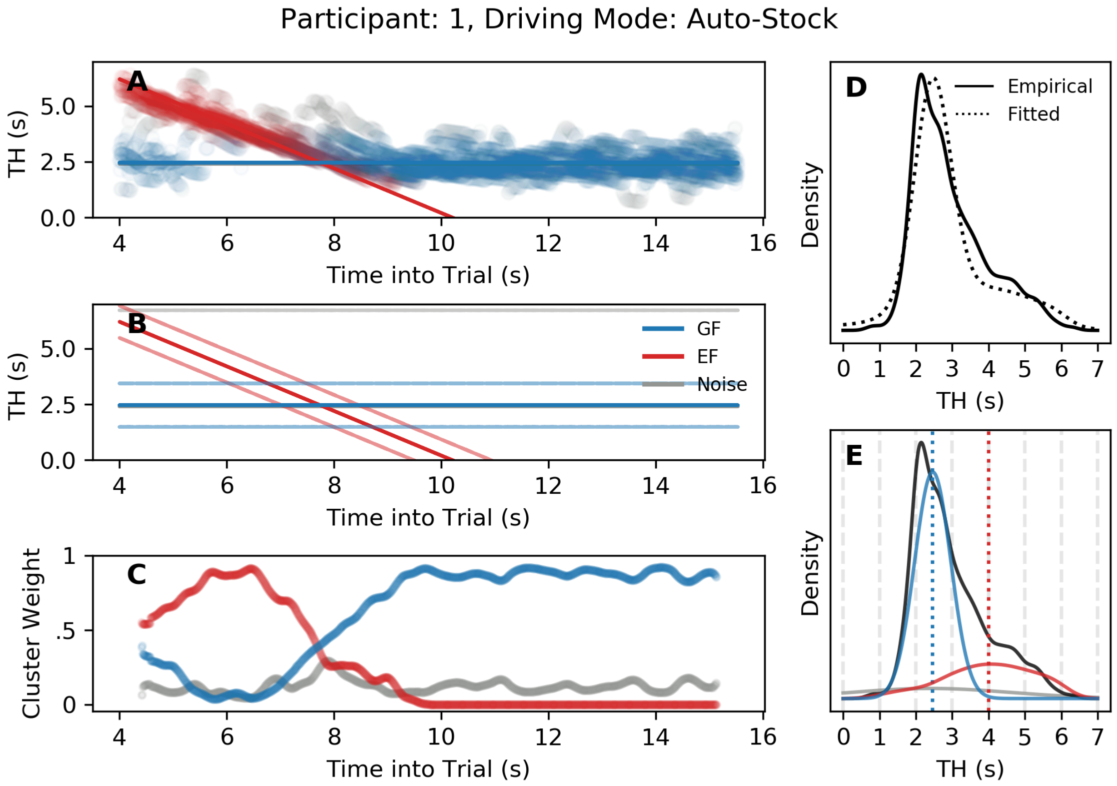

SI Fig 26

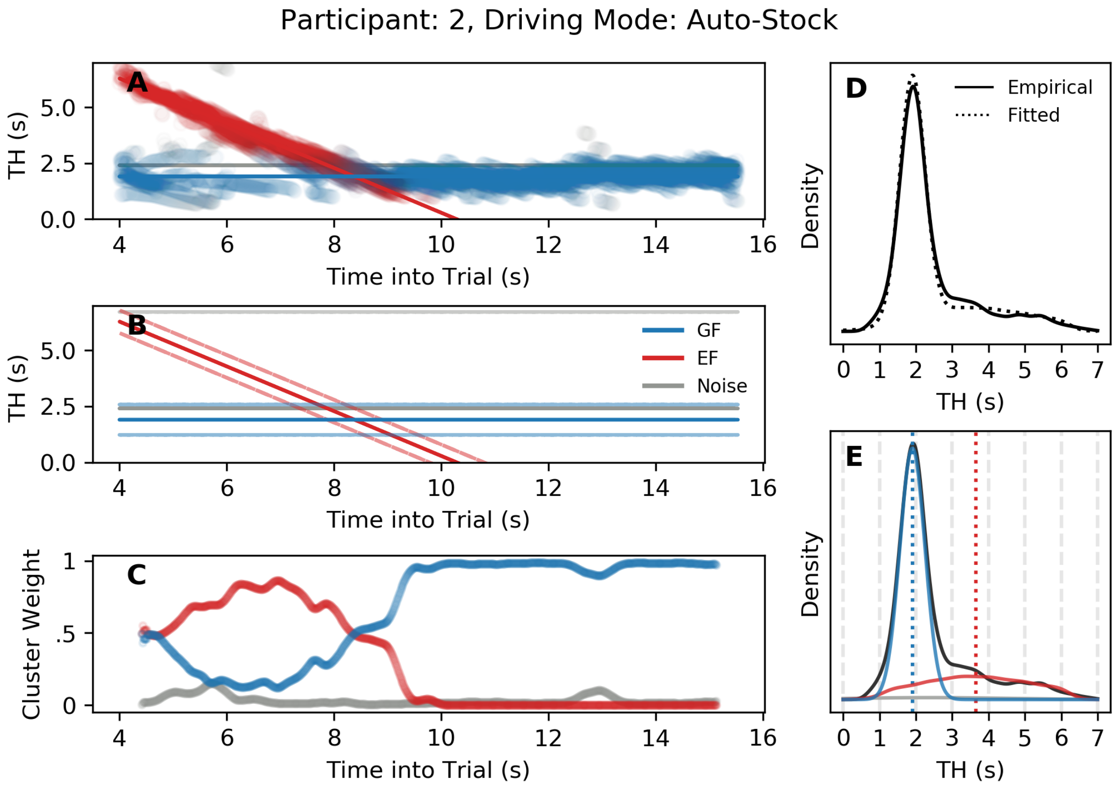

SI Fig 27

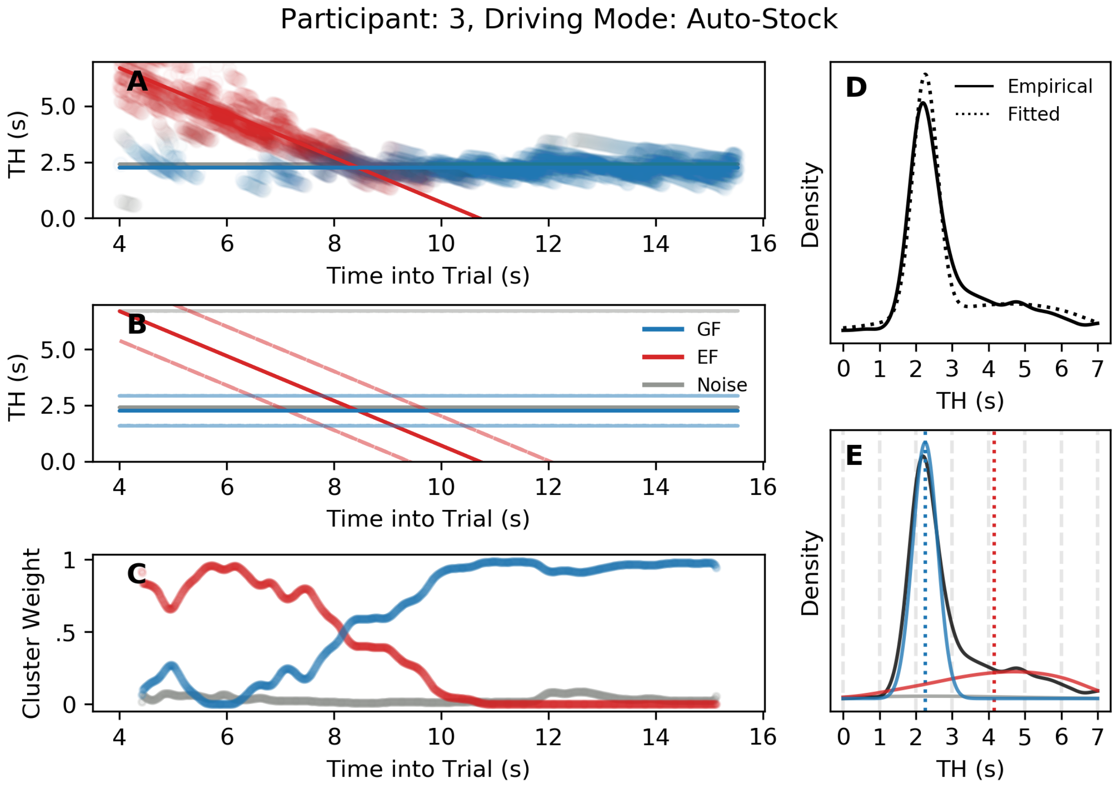

SI Fig 28

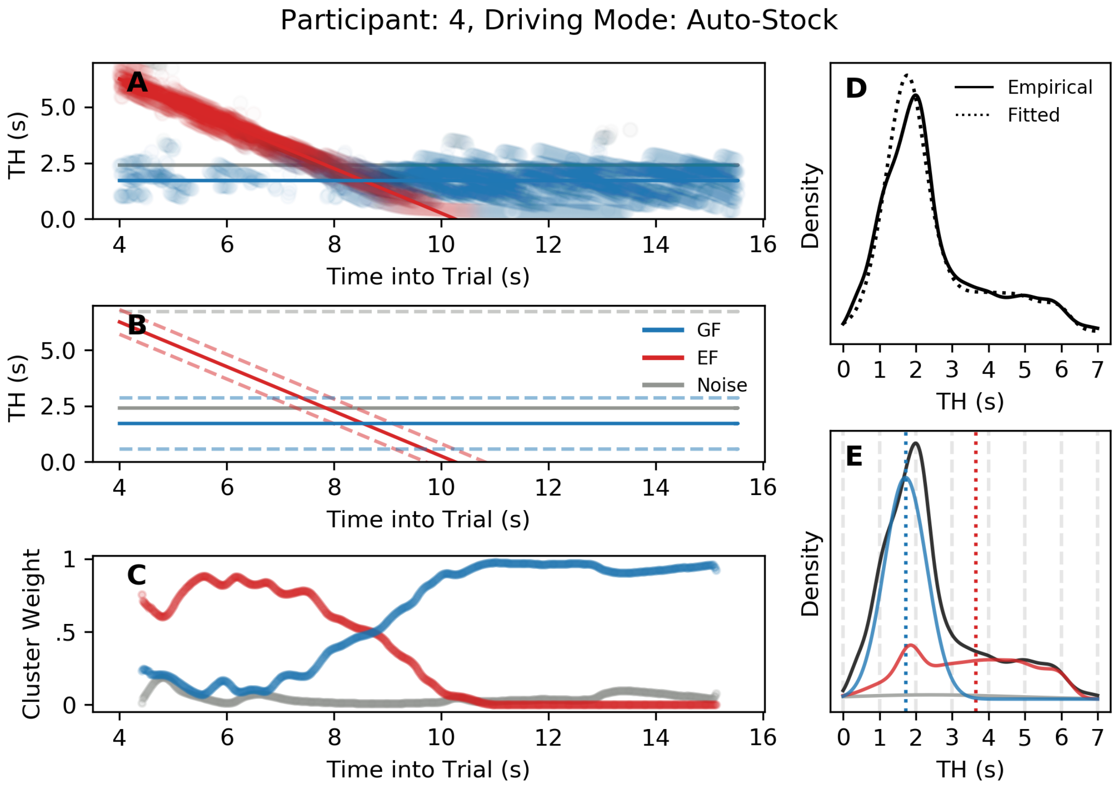

SI Fig 29

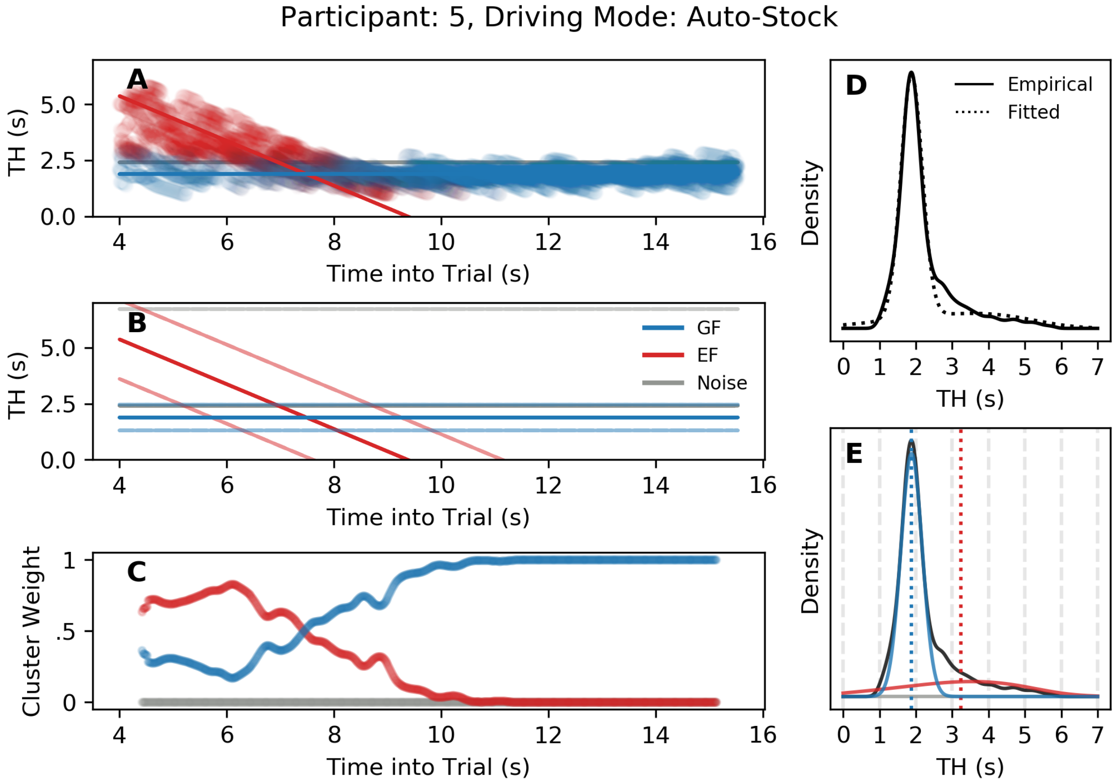

SI Fig 30

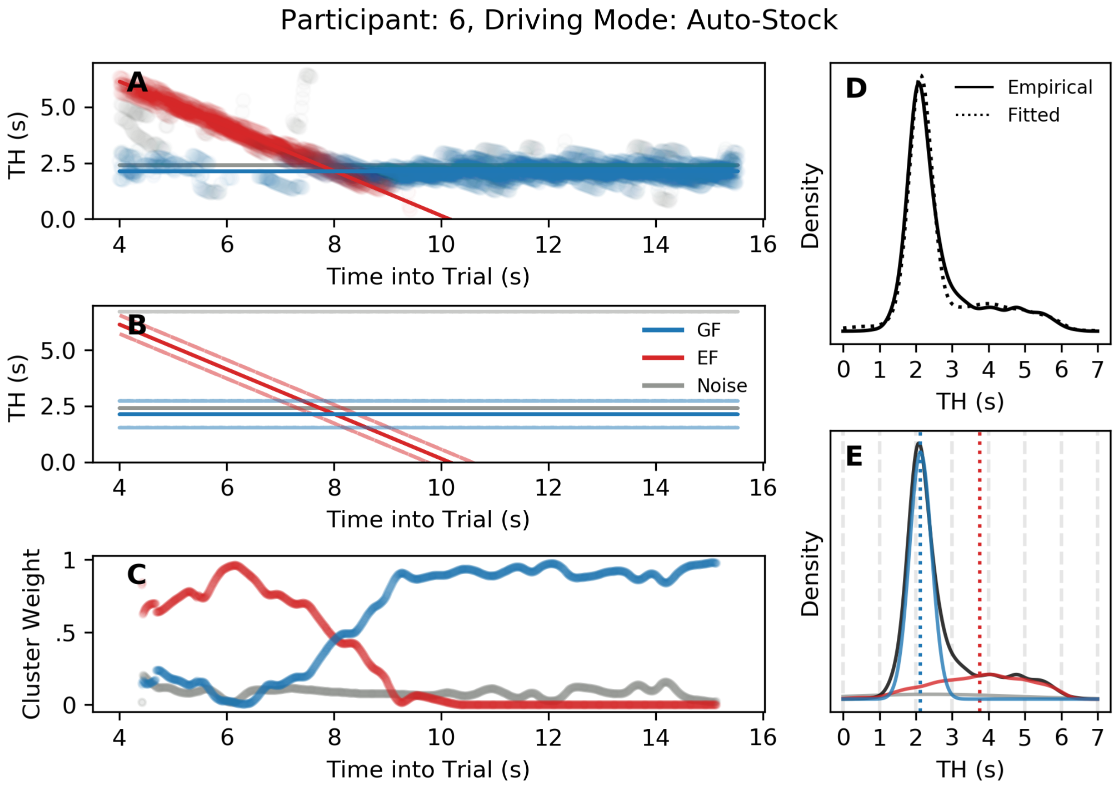

SI Fig 31

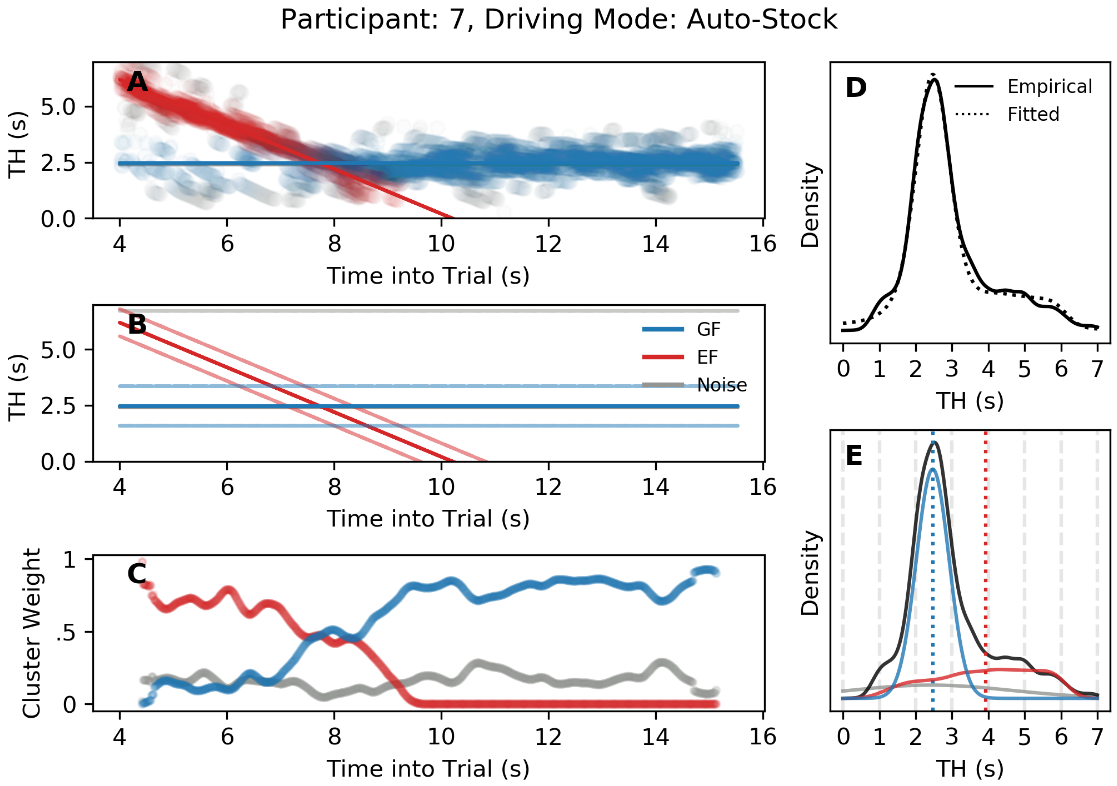

SI Fig 32

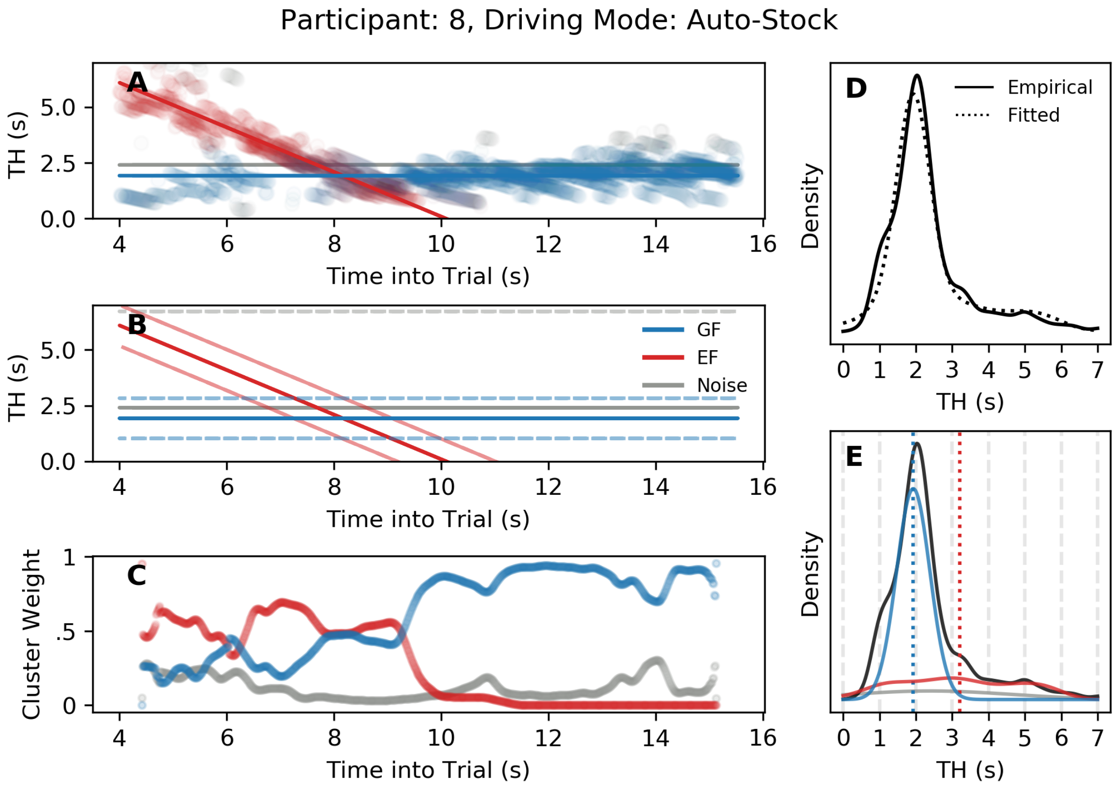

SI Fig 33

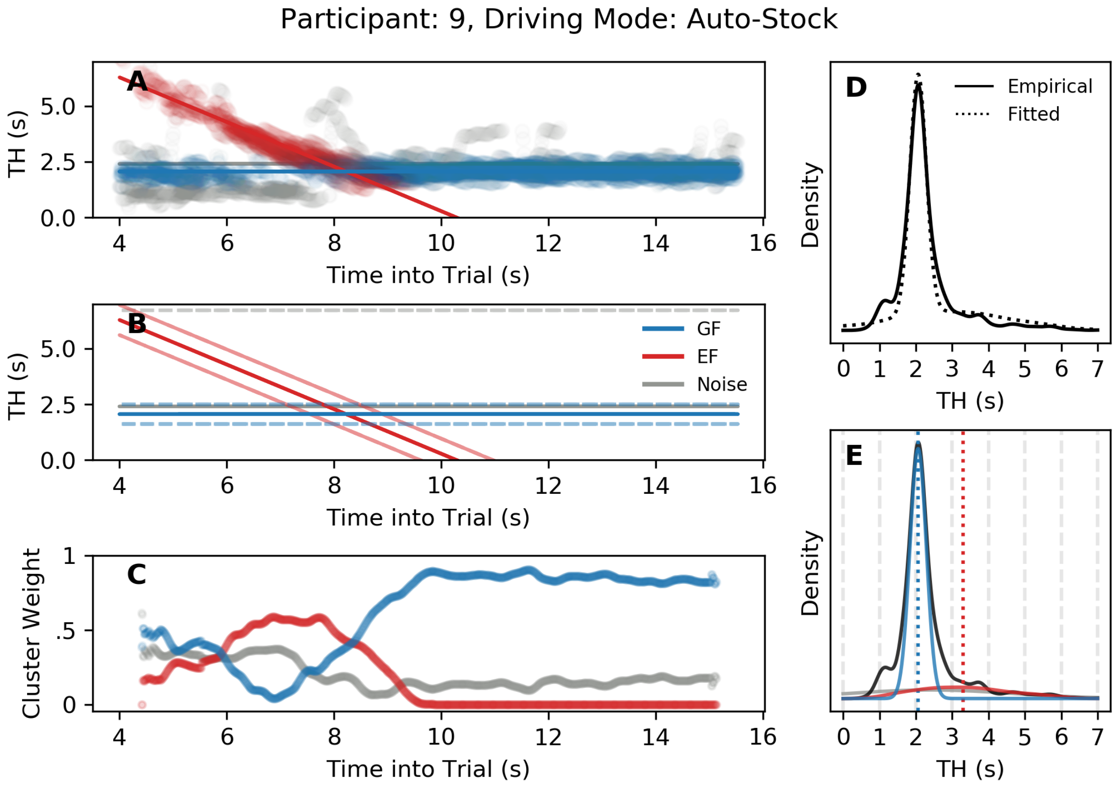

SI Fig 34

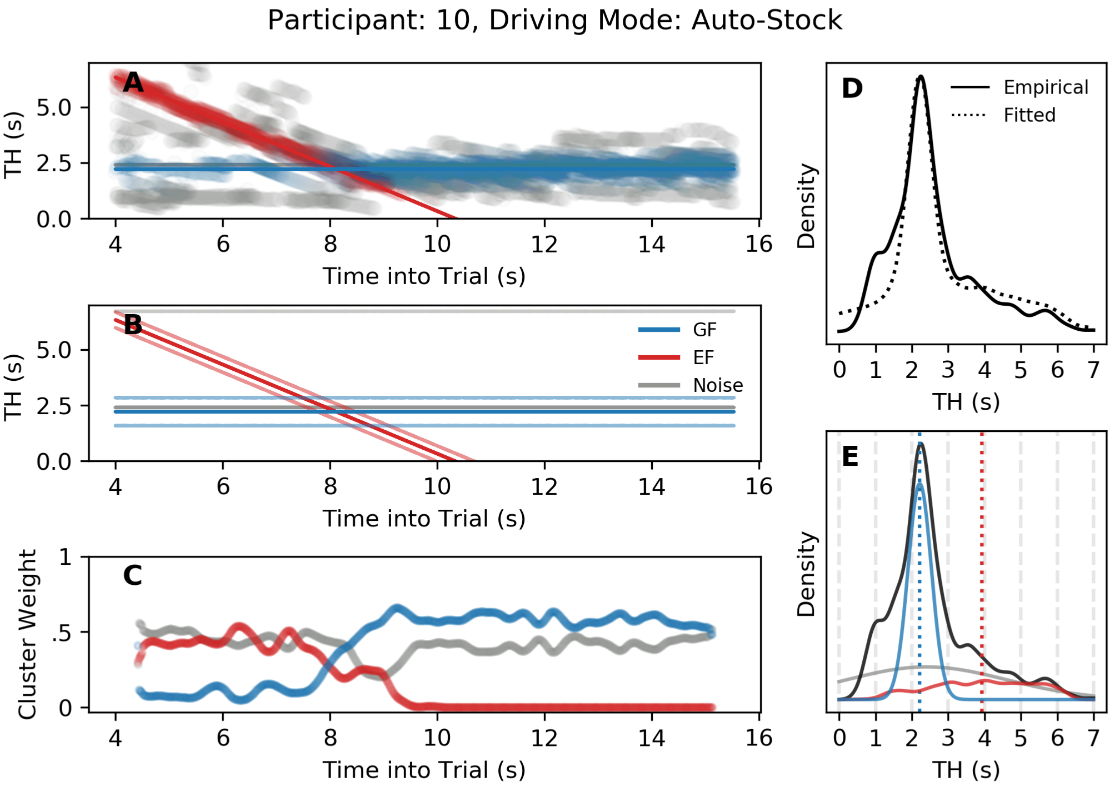

SI Fig 35

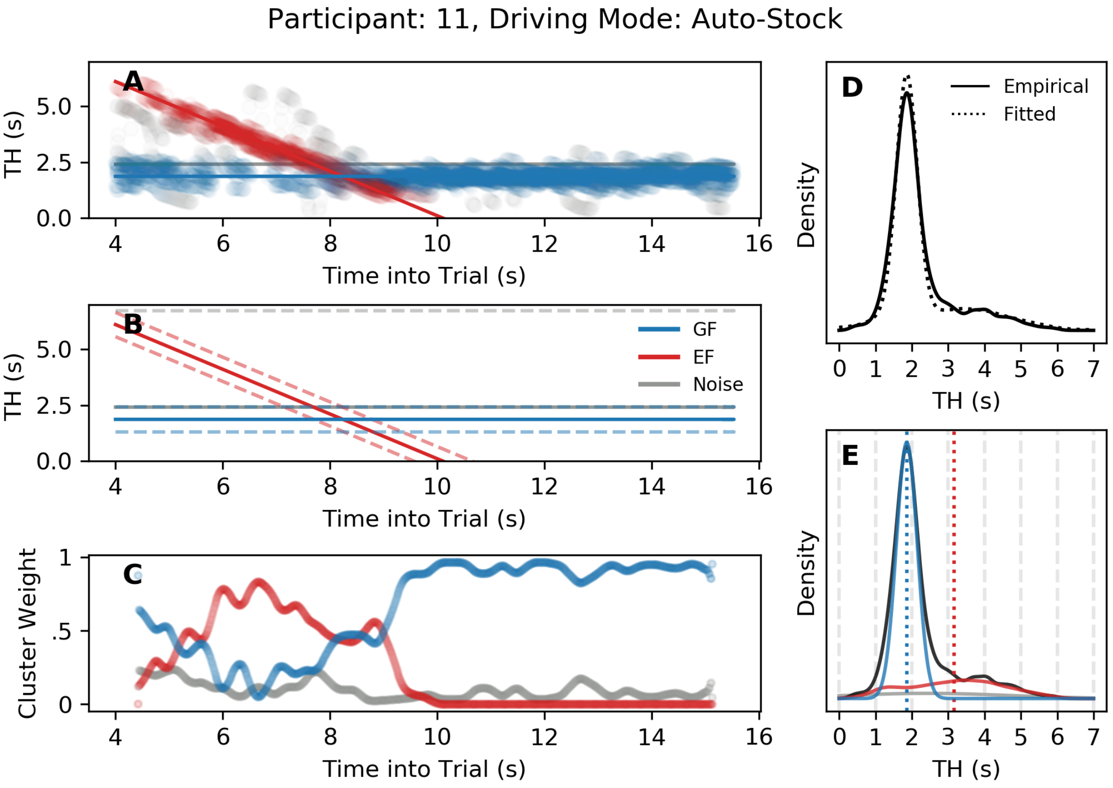

SI Fig 36
